# Assessing Trauma History in Pregnant Patients: A Didactic Module and Role-Play for Obstetrics and Gynecology Residents

**DOI:** 10.15766/mep_2374-8265.10925

**Published:** 2020-07-20

**Authors:** Natalie R. Stevens, Lucie Holmgreen, Stevan E. Hobfoll, Jamie A. Cvengros

**Affiliations:** 1 Assistant Professor, Department of Psychiatry & Behavioral Sciences, Rush University Medical Center; 2 Assistant Professor, Department of Psychological Science, Gustavus Adolphus College; 3 Founder, STAR: Stress, Anxiety and Resilience, LLC; 4 Associate Professor, Department of Psychiatry & Behavioral Sciences, Rush University Medical Center

**Keywords:** Obstetrics, Trauma Sensitive, Screening, PTSD, Post-Traumatic Stress Disorder, Pregnancy, Communication, Intimate Partner Violence, Women's Health, Neonatal-Perinatal Medicine, OB/GYN

## Abstract

**Introduction:**

Assessing and addressing patient histories of trauma constitute a critical component of care for vulnerable populations such as pregnant patients, yet they often go unrecognized in obstetric care. Obstetric providers may feel poorly equipped to address this issue comfortably and effectively.

**Methods:**

We designed this didactic module for obstetric residents with previous experience taking patient histories and delivering clinical care. The module was delivered with a faculty member and three additional facilitators with expertise in communication skills training. The session included 60 minutes of background information followed by a 15-minute presentation of a communication template for assessing trauma history. Using a practice case, residents had 45 minutes to practice in small groups, with the facilitators serving as the patient in the role-play.

**Results:**

In the 2015-2016 academic year, 21 obstetric residents participated in this module. All residents (100%) endorsed favorable beliefs regarding the importance of assessing trauma history and using trauma-informed care. On average, three-fourths (77%) demonstrated basic awareness of issues related to trauma in medical populations. Most residents (>85%) reported a high sense of efficacy in delivering trauma-informed care in some areas, while fewer (64%) reported efficacy specifically in educating patients about signs and symptoms of traumatic stress.

**Discussion:**

The module was well received overall, with participants indicating that clinical training in trauma-informed communication was needed to enhance their sense of efficacy in this area. This learning exercise provided training in a critical communication skill while highlighting areas for further development.

## Educational Objectives

By the end of this activity, learners will be able to:

1. Identify the potential impact of traumatic stress on pregnancy outcomes.
2. Identify common barriers to effectively assessing trauma history in pregnant patients.
3. Utilize empirically supported approaches to assess a patient's trauma history and respond to disclosures.

## Introduction

Assessing histories of trauma in patients who are pregnant is an essential component of preventive care in order to identify risk factors for adverse perinatal outcomes.^1-5^ The American College of Obstetricians and Gynecologists (ACOG) recommends that health care providers routinely screen all women for history of sexual assault, for example, in an effort to prevent and mitigate any long-term persistent medical and psychological consequences of these traumatic experiences.^6^ Research demonstrates that although OB/GYN residents believe in the importance of assessing and addressing their patients’ histories of trauma, abuse, and interpersonal violence, most feel unprepared and insufficiently trained to effectively take on the task.^7^

Some curricula exist to fill gaps in communication training related to patients experiencing pregnancy complications and difficult topics such as pregnancy loss.^8^ However, communication skills curricula focused on traumatic stress or assessing trauma history are less common or are specific to veteran populations.^9,10^ Based on a recent search in *MedEdPORTAL,* no trauma-informed communication skills modules exist that specifically apply to the context of pregnancy. Moreover, in order to fully appreciate how historical traumatic experiences can exert ongoing adverse effects on the health of pregnant women and their developing fetuses, trauma-informed communication must capture more than just acute crises such as current intimate partner violence.^11^

Thus, this module has been developed to provide background knowledge on the potential adverse effects of traumatic stress on pregnancy outcomes as well as a structured, empirically supported framework for taking a trauma history and responding effectively and empathically to disclosures of trauma. This activity is intentionally targeted toward OB/GYN residents to provide them with a safe learning environment in which to practice skills that they can immediately apply to their day-to-day clinical environments in the management of obstetric patients. Although specific instructions for how to implement principles and practices of trauma-informed care are beyond the scope of the module, references in it (e.g., the facilitator guide, Appendix A; the PowerPoint slides, Appendix B) include links to relevant research articles and resource such as the ACOG committee opinion on sexual assault^6^ and the Substance Abuse and Mental Health Services Administration guide to trauma-informed care for guidance and consultation.^12^

This module has been designed to offer initial training in taking a patient's trauma history and gathering pertinent information regarding ongoing psychological distress associated with trauma. The module also includes a simulation or role-play exercise in which to practice these skills alongside peers. The module was developed and piloted with OB/GYN residents because they are primary caregivers of pregnant patients in academic medical settings. Given the particular risks associated with traumatic stress in perinatal populations, we also felt that the training fit within the defined scope of obstetrics practice. However, the module is sufficiently introductory that it would be appropriate for students/trainees in general medicine, nursing, midwifery, and physician's assistant programs. Prerequisite knowledge of traumatic stress may be helpful but is not necessary. Optimally, residents or other learners encountering this module would have achieved the basic foundations of effective patient communication, demonstrating competency in active listening, empathic reflection, and responding with nonjudgmental acceptance.

The framework for taking the patient's trauma history and responding to disclosures has been developed using empirically supported approaches.^13,14^ These approaches utilize behaviorally anchored phrases in an effort to avoid terminology with variable meanings (e.g., favoring use of the term *forced sexual activity* vs. *rape*). Thus, the framework reduces the likelihood of false-negative responses to the screening while also minimizing stigma associated with experiences of sexual violence. The standardized role-play exercise is designed to provide the opportunity to practice empathic communication utilizing these behaviorally anchored phrases.

## Methods

### Target Audience

We designed this module for OB/GYN residents at any year of residency. The existing residency curriculum at our institution included didactic lectures on mental health screening and medical management of depression during the perinatal period. However, the curriculum did not include advanced communication skills training in mental health, traumatic stress, or trauma-informed care. Residents were expected to have a basic knowledge of mental health issues affecting the perinatal period but no prior knowledge specifically about trauma. Therefore, this module and its standardized role-play exercise were designed to serve as an introduction to communication with pregnant patients having histories of trauma and possibly also active symptoms of traumatic stress.

### Didactic Module

#### Facilitator training:

Facilitators for this module included one faculty leader and three instructors in behavioral sciences. The faculty leader, a clinical psychologist, had 5 years of clinical experience in the assessment and treatment of trauma-related disorders in perinatal populations as well as 4 years of experience teaching as part of an advanced communication skills curriculum for medical students. The three instructors were clinical psychology postdoctoral fellows in the process of completing a 2-year postdoctoral fellowship in traumatic stress and women's health psychology. One of the instructors also taught in the advanced communication skills curriculum for medical students. Thus, all facilitators had background in the screening, assessment, and psychological treatment of trauma-related disorders in perinatal populations, and two of the four had experience teaching communication skills to medical trainees.

Facilitators participated in the development of module materials, including collecting scientific literature on traumatic stress and pregnancy, *Diagnostic and Statistical Manual of Mental Disorders, Fifth Edition* criteria for post-traumatic stress disorder (PTSD), empirically supported methods for assessing and responding to trauma histories, and case material development for the role-play exercise. Facilitators met as a group with the faculty leader to construct the didactic module, develop the case materials, and rehearse the role-play exercise.

The faculty leader also provided training to facilitators in offering feedback to OB/GYN residents when they participated in the standardized case role-play. Feedback training covered providing specific behavioral examples of effective versus less-effective communication around the topic of trauma and addressing and providing support for discomfort/distress residents might encounter upon practicing these skills. As clinical psychologists, facilitators were encouraged to utilize their expertise when addressing residents’ discomfort or significant distress.

#### Space requirements:

For our OB/GYN program, each class (by year) contained six residents, for a total of 24 residents. Rather than delivering the module to each class separately, we opted to deliver it to the entire program of 24 residents. This saved time while also allowing more-senior residents to share their experiences and expertise with first- and second-year residents. The module was delivered as a onetime stand-alone didactic over 2 hours. The classroom space utilized was a traditional classroom setting where the residents’ didactics usually took place, with desks and chairs for 24 residents as well as the space to break out into small groups for the standardized case role-play exercise. Thus, the classroom needed to be large enough for the group of 24 residents to be comfortably divided into groups of four to five participants, with enough distance between the small groups so that they did not distract each other.

#### Time requirements:

The didactic module was designed to last 2 hours on the following schedule:

• 7:30 am-8:30 am: background, didactics, and discussion in the full group of 24 residents.
• 8:30 am-8:45 am: presentation of communication template for taking the trauma history.
• 8:45 am-9:30 am: small-group practice (four to five residents) with a designated person serving as the patient (a facilitator, provided there were enough facilitators) followed by wrap-up.

#### Materials needed:

Necessary equipment included a projector and screen for the didactic portion of the module.

#### Documents needed:

The following documents were needed for the didactic portion and the standardized case role-play portion of the module:

• Didactic facilitator guide (Appendix A).
• PowerPoint slides (Appendix B) for background on the didactic.
• Handout 1: sample chart of a pregnant patient with PTSD (Appendix C).
• Handout 2: communication template for taking a trauma history (Appendix D).
• Handout 3: sample trauma-informed practice (Appendix E).
• Handout 4: sample trauma narrative for the role-play (Appendix F).
• Pocket guide for trauma history screening (Appendix G).

#### Module delivery:

This module was delivered to 21 OB/GYN residents (three residents were absent that day) in academic year 2016-2017. Four facilitators (one licensed clinical psychologist and three clinical psychology postdoctoral fellows) conducted the didactic module following the facilitator guide (Appendix A), PowerPoint slides (Appendix B), and handouts (Appendices C–E). Following presentation of the background and rationale for assessing trauma history in pregnant patients, the facilitators prompted a brainstorming discussion of common barriers providers experience that get in the way of asking patients about their past traumas. The purpose of this brief discussion was to engage residents in thinking about experiences caring for pregnant patients, as well as the challenges they have faced, and to normalize these challenges while guiding the discussion toward effective communication solutions. The purpose was not necessarily to generate an exhaustive list or a solution to every barrier but rather to introduce communication materials addressing three common barriers frequently cited in research: time, lack of communication training (i.e., what to say vs. what not to say), and fear of retraumatizing patients.^15,16^ Following the discussion of barriers, facilitators presented and described the communication template for taking a trauma history and responding to disclosures. The communication template formed the basis for the role-play exercise in which residents had the opportunity to put communication skills into practice.

The four facilitators then led the role-play exercise. All residents participated in the role-play exercise in groups of four or five, with one facilitator serving as the patient using the sample trauma narrative (Appendix F). In small groups, residents were expected to use specific communication skills provided in the communication template (Appendix D) and in the sample trauma-informed questions, phrases, and statements for practice (Appendix E) to assess and effectively respond to the patient's history of trauma and how it was affecting her in her current pregnancy. Residents were provided with a broad-strokes guide to assessing a trauma history (Appendix G) for use in the role-play exercise.

The standardized case role-play exercise was conducted using a round-robin interview style whereby each resident took turns assessing the patient's trauma history and responding to the patient's answers. Residents were expected to engage in this exercise using the sample trauma-informed phrases and responses, which communicated empathy, sensitivity, and nonjudgmental acceptance of the patient's history. Facilitators provided on-the-spot feedback to residents and led the small groups in a brief discussion of what aspects of the interview went well and what aspects were most challenging for residents. Residents were encouraged to offer one another constructive feedback, with facilitators providing additional feedback regarding aspects of the patient's responses that could have been better followed up and aspects that warranted empathic acknowledgment and possible referral for counseling.

During the didactic session, residents were invited, using open-ended prompts, to openly share comments and reflections about providing care for patients with histories of trauma. Residents were also invited to discuss what could be improved in their residency training to help them feel better prepared and more effective at talking with their patients about trauma and managing traumatic stress. Responses were intended to demonstrate informal feedback based on residents’ experience of the didactic module. A facilitator recorded verbal statements that residents made during the session and immediately following the session one-on-one with a facilitator. Comments and reflections from residents who sent emails later, after the session, were also saved for this purpose. These comments and reflections were compiled by the faculty leader to serve as feedback on the module.

### Assessment Tool

Following the didactic and role-play module, residents had the opportunity to complete an anonymous assessment of their beliefs, level of awareness, and sense of efficacy related to the delivery of trauma-informed care. The purpose of this assessment tool was to gather information about residents’ level of awareness and sense of efficacy in treating pregnant patients with histories of trauma. Items on the assessment tool were slightly adapted from an existing research measure designed to assess trauma providers’ awareness, views, and practice of trauma-informed medical and nursing care.^15,16^ The assessment tool was distributed to residents via email link to an electronic survey utilizing the REDCap platform. A copy of the assessment tool is provided in Appendix H.

Data collected from this assessment of OB/GYN residents and presented here are intended to guide future adaptations of this trauma-informed OB/GYN didactic focused on assessing and addressing trauma histories in pregnant patients. Responses collected from residents who participated in this module have also been included in a larger research study of medical providers’ personality traits and sense of efficacy in delivering trauma-informed care.^17^

Finally, during a session debrief following the didactic, comments from facilitators were collected about what went well and what could be improved for future sessions. Facilitators also commented on direct observations made during the role-play, given that the faculty leader and all facilitators were able to observe only their own small groups during the role-play.

The Rush University Medical Center Institutional Review Board granted ethical approval for the research procedures described in this report under exempt status.

## Results

According to Kirkpatrick's pyramid,^18^ we conceptualized the evaluation of the impact of our module in terms of residents’ reactions to participating in the module and in the learning that they demonstrated following the module. The role-play exercise offered the most robust opportunity in terms of observing and gauging residents’ comfort with the learning materials, using terminology in the communication template, and responding to the patient's answers provided in the trauma narrative. Navigating the how-to process of asking about trauma, what to say, and what not to say appeared to be the most challenging for residents; hence, facilitators felt that role-play was probably the most useful learning strategy.

Facilitators observed the role-play and provided feedback regarding aspects of the patient's responses that might warrant follow-up questioning or empathic acknowledgment. For example, when the patient reported pain with sex and said she “goes somewhere else in her head,” facilitators reinforced that this might signal a need for following up on pain and associated symptoms, as well as for reflecting the patient's response with empathy, by saying, for example, “It sounds like there are some difficulties/issues you are having with sex with your boyfriend that you try to distract yourself from. It sounds like that might be hard/unpleasant for you. Would you like to tell me more about that? There might be something we can try to do to help with the pain.” Facilitators also offered feedback on potential opportunities to provide information or referrals for counseling, again using empathic reflection to approach the topic. For instance, when the patient said she was “fine” and did not need mental health treatment but acknowledged that past abuse meant that she avoided certain family members, a possible response could have been “It can be difficult when you feel like you have to change your actions because of something someone else did. It affects your life,” or “Experiences with abuse can affect people's lives in different ways. Sometimes counseling can help with issues like family/partner relationships. Let me know if you would ever like more information, we can always talk more about it.” Facilitators emphasized that the goal in the moment was not necessarily to guide the thread of conversation to fully examining and solving a problem that might be related to past trauma. Rather, the goal was to open the door to further dialogue by asking a few open-ended questions and responding empathically, thereby increasing the likelihood that the patient would feel safe to share physical, social, and emotional trauma-related problems to the extent that they were affecting her current functioning. These issues could be set aside to address at a later time; however, facilitators emphasized that patients were more likely to feel comfortable engaging in such a conversation when the initial assessment and response were clear, nonjudgmental, and empathic.

Additionally, we assessed residents’ sense of efficacy in delivering trauma-informed care to their pregnant patients in obstetrics settings. Although skill competence was not itself a specific learning objective of this module (i.e., residents were not formally assessed on their skill in taking a trauma history and responding to the disclosure), perceived sense of efficacy ratings were deemed informative in guiding future development of trauma-informed training for OB/GYN residents. We further summarize the findings from our learning assessment and qualitative feedback (reactions) below. We also summarize reactions of the facilitators in conducting this module with OB/GYN residents.

### Comments From Residents (Learners)

Comments and reflections from residents recorded during or immediately after the module or later in writing to the faculty leader revealed a number of important findings. Residents reported that this topic was highly relevant to their clinical practice given the number of patients they treated who were affected by trauma. While remarking that these skills did not fully address their concern for managing a patient with current or ongoing trauma, residents acknowledged that they could be applied to a variety of clinical situations in which a patient struggled with traumatic stress and needed additional support. Via email, one learner wrote that addressing how treating patients with trauma could personally affect residents sparked an interest in seeking support for preventing the learner's own secondary traumatic stress/vicarious trauma. The lead faculty member responded to this email, validating the learner's experience and applauding their awareness of these emotional responses, as well as inviting them to use staff psychologists as a confidential resource for additional tools and support. The faculty leader did not probe for more specific details from the learner but normalized their feelings and the importance of bringing challenging experiences to trusted mentors, peers, and other institutional support services. Specific comments/reflections from residents are listed below:

• Comments during didactic portion:
  ○ “I was really worried about having to [listen to] all the graphic details of the patient's trauma. But once you use the skills you realize that's not what this is about.” [senior resident]
  ○ “I had a patient who was sexually assaulted. I just used one of the phrases on this worksheet and I saw how she immediately relaxed.” [senior resident]
  ○ “We are the ones who do the pelvic exams. If the patient is anxious or upset, it's our job to help them or we could make things worse.” [junior resident]
  ○ “You can just tell the patient that if they have any of these things [past traumas] it affects their health. That way you show you aren't prying.” [senior resident to junior resident]
• Comment during role-play exercise:
  ○ “I feel like I don't know what to say to these patients. I really need help with this.” [junior resident]
• Email communication following didactic:
  ○ “It definitely brought me to reflect on some ‘stuff’ that I face on a day-to-day basis. [Separating work and personal life] is nearly impossible when I am treating a patient affected by past illness/trauma. What resources can I look up for myself and my co-residents to help cope with these feelings?” [junior resident]

### Assessment Tool Responses

A total of 14 OB/GYN residents (67% response rate) completed the assessment tool on beliefs, awareness, and sense of efficacy in delivering trauma-informed care. Results of the responses to the assessment tool are presented in the Table. Most residents (85%-100%) responded with favorable beliefs regarding the benefits of assessing and addressing histories of traumatic stress in their patients. Of the 11 awareness items on the assessment tool, four had specified correct answers based on previous research demonstrating adequate knowledge or awareness of key issues of trauma-informed care.^15,16^ The proportion of residents providing correct answers to these questions was variable, as shown in the Table. All residents who completed the assessment knew that patients with post-traumatic stress reactions do not always show obvious signs of distress, while slightly more than half knew that developing PTSD was not inevitable following exposure to trauma. Residents responded that they felt the most efficacious in responding calmly to a patient's emotional distress, explaining the steps of an exam ahead of time, and providing feedback to patients during exams and procedures. Residents responded that they felt the least efficacious in educating patients about common traumatic stress reactions and symptoms.

**Table.**
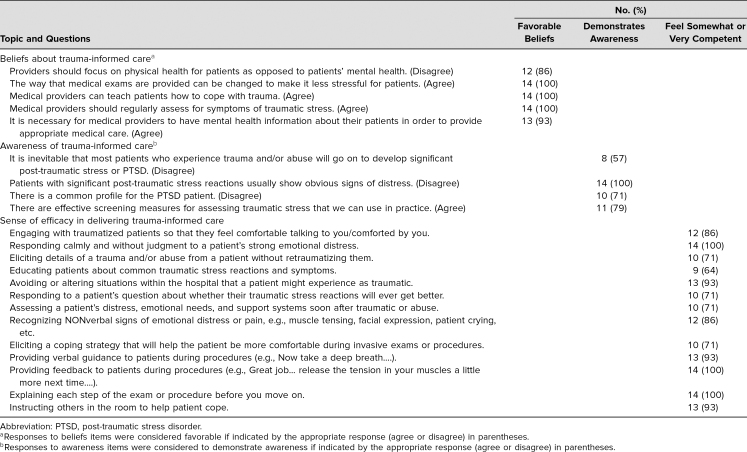
Results of Residents’ Beliefs, Awareness, and Sense of Efficacy in Trauma-Informed Care (*N* = 14)

### Comments From Facilitators

Facilitators commented that this module addressed an important topic that most residents felt they were insufficiently trained to address effectively. Facilitators observed some defensiveness on the part of a few residents upon receiving feedback or being instructed to ask about trauma histories directly. However, they noted that most residents were extremely grateful to have resources describing concrete steps toward eliciting sensitive information that could affect a patient's health and specific examples of how to respond when a patient endorsed having a history of past traumatic events. Observations and suggestions for improvement from facilitators included the following:

• Observations:
  ○ “The residents seemed to really hunger for information and the specific ‘how-to’ as well as the opportunity for practice and to get expert feedback.”
  ○ “Having senior residents model communication for junior residents was useful because they often had good case examples of when skills were used effectively, but occasionally senior residents did not provide the most effective or helpful modeling and may still need to be corrected/shaped.”
  ○ “There was some defensiveness observed on the part of some residents who resisted engaging in the roleplay and use of direct/explicit trauma language.”
• Most useful: emphasize role-play/practice/concrete tools:
  ○ “Most useful was actual demonstration and engaging them in practice.”
  ○ “Spend more time on demonstration of communication skills, practice, and providing feedback.”
  ○ “Medical providers seem to really benefit from/need practical tools such as the laminated cards with critical steps as a reminder.”
• Less useful: lecture/didactic/extensive background:
  ○ “If time is limited, reviewing diagnostic criteria for PTSD is not necessary.”
  ○ “Spend less time on lecture/didactics or keep very simple and concrete.”

## Discussion

The purpose of this module was to give obstetric residents the knowledge, framework, concrete steps, and safe environment to practice assessing histories of trauma in pregnant patients. The rationale for developing this module was twofold: Trauma history is considered a risk factor for adverse outcomes of pregnancy, and ACOG recommends screening and appropriate response to histories of abuse, violence, and trauma among patients who are pregnant.^6^ Moreover, curricula specifically designed to address the learning needs of obstetric residents were found lacking in the literature. The module was introduced into the OB/GYN residency curriculum in academic year 2016-2017. At the end of the module, learners were expected to (1) identify symptoms of traumatic stress and the potential impact of traumatic stress on pregnancy outcomes, (2) identify common barriers to effectively addressing traumatic stress in obstetrics settings, and (3) utilize empirically supported approaches to assess a patient's trauma history and respond to disclosures. These objectives were achieved through a 2-hour combined didactic lecture and skills demonstration and practice delivered by experienced educators.

Overall, the module was successful in meeting students’ needs and our proposed objectives. The 2-hour didactic was well organized and well received. The allotted time was deemed sufficient to provide a basic overview of traumatic stress and its manifestations in pregnancy, concrete guidance on how to conduct an empirically supported assessment of trauma history, and a chance to conduct skills practice with residents and their peers. We believe this module provides a clear, organized, and effective learning experience with an appropriate level of content and opportunity for practice for obstetric residents.

In reviewing the results of our assessments and feedback from both facilitators and learners, we identified four limitations, which offer opportunities for future improvement or expansion of the module in its current form. First, our assessment of awareness and sense of efficacy in delivering trauma-informed care highlighted potential areas for future focus. Residents demonstrated awareness gaps in understanding the likelihood of developing PTSD following trauma, which could be associated with overpathologizing or underpathologizing patients who are survivors of trauma. Residents also reported lower sense of efficacy involving counseling patients about traumatic stress symptoms and treatment and helping patients identify specific coping strategies to minimize trauma triggers in the obstetric environment. It may be useful to expand the content of the module either through in-session learning or premodule readings and materials on the topic of traumatic stress. Although the focus of this module was assessment of trauma history and responding to disclosures, residents may feel more confident performing this task if they have a more-solid understanding of the effects of trauma and psychopathological responses to trauma. Helping patients to identify specific coping strategies to deal with active trauma symptoms in the context of obstetric care was also not the focus of this module; however, it is undoubtedly important. A future iteration of the module could include addressing trauma symptoms and managing triggers, for example, during invasive exams and procedures or in preparation for potentially retraumatizing experiences such as childbirth, obstetrical surgeries, and obstetrical emergencies.

Second, it was observed that having senior residents practicing alongside junior residents was helpful in some, but not all, cases. Senior residents have more experience to draw from, but this did not always lead to effective modeling for junior residents. It may present a challenge to teach to different levels of training in one 2-hour didactic session. One possible opportunity for improvement could be to deliver the module to smaller groups of residents based on their training year, tailoring practice according to the groups’ varying levels of clinical experience.

Two additional limitations concern the assessment methods used to evaluate the module. Although we present information regarding learner feedback and reflections, formal qualitative assessment methods such as focus groups, structured interviews, or evaluation forms were not used. Comments from participants may have been biased in that they may have come only from participants with favorable views of the module or of the necessity for trauma-informed care. A final limitation concerns the lack of tests of the effectiveness of the module. The assessment tool was distributed only after the module and therefore does not reflect pre-post change in any of the topics assessed. Additionally, due to time constraints, the module did not involve an opportunity to formally test knowledge and skills in a formal standardized patient encounter. Moreover, assessment of residents’ sense of efficacy cannot be assumed to be a reliable measure of actual competence. It is unknown how the information and practice residents received translated into practice outside the classroom setting. It would be useful to determine the effectiveness of the module using the included standardized patient case in a formal simulation setting where learners have the opportunity to practice and receive feedback from standardized patients as well as feedback from peers and program instructors.

Notwithstanding these limitations, our results demonstrate that this education activity can fill an important gap in OB/GYN resident communication skills learning, providing knowledge, tools, and a safe environment in which to practice asking patients about a highly sensitive topic (trauma history). The reactions of some participants suggest that they have begun to reflect on and apply the skills. One response from a resident also prompted us to include a specific acknowledgment of how discussing trauma can raise issues related to the learners’ own trauma/secondary trauma, and future use should include an acknowledgment of this along with relevant referral resources. Although designed and examined in obstetric residents who had already acquired clinical training and experience with medical patients, the module and its contents are sufficiently introductory to be suitable for students/trainees in other programs, such as general medicine, nursing, midwifery, and physician's assistant programs. Given the prevalence of traumatic stress and the associated risks of adverse medical outcomes across various medical populations and settings, we believe that modules such as ours could be integrated into trauma-informed care initiatives at the organization level. These materials can be seen as a step toward creating trauma-informed systems where members at all levels of the health care organization (not just clinicians) are equipped to identify traumatic stress and respond effectively.

## Appendices

Didactic Facilitator Guide.docxPowerPoint Slides.pptxHandout 1 Sample Chart of Pregnant Patient With PTSD.docxHandout 2 Communication Template.docxHandout 3 Sample Trauma-Informed Practice.docxHandout 4 Sample Trauma Narrative for Role-Play.docxPocket Guide for Trauma History Screening.pdfAssessment Tool.docx
All appendices are peer reviewed as integral parts of the Original Publication.
