# Supplementary material for: Assessing Trauma History in Pregnant Patients: A Didactic Module and Role-Play for Obstetrics and Gynecology Residents
Source: MedEdPORTAL. 2020 Jul 20;16:10925. doi: 10.15766/mep_2374-8265.10925 (PMC7373354; doi:10.15766/mep_2374-8265.10925)
Supplement: Supplementary file 1 — Didactic Facilitator Guide.docxPowerPoint Slides.pptxHandout 1 Sample Chart of Pregnant Patient With PTSD.docxHandout 2 Communication Template.docxHandout 3 Sample Trauma-Informed Practice.docxHandout 4 Sample Trauma Narrative for Role-Play.docxPocket Guide for Trauma History Screening.pdfAssessment Tool.docx [file mep_2374-8265.10925-s001.zip › A. Didactic Facilitator Guide.docx]

**trauma-informed obstetric Care**

**OB/GYN Resident Didactics Outline**

**Objectives for Trauma-informed Obstetric Care Didactic**:

1. Identify the potential impact of traumatic stress on pregnancy outcomes
2. Identify common barriers to effectively assessing trauma history in pregnant patient
3. Utilize empirically supported approaches to assess a patient’s trauma history and respond to disclosures.

**Materials/Handouts Needed for Didactic:**

1. PowerPoint slides to show definitions and background information (Appendix B).
2. Sample chart of pregnant patient with trauma history (Appendix C).
3. Communication template for taking a trauma history and responding to disclosures (Appendix D).
4. Sample trauma-informed questions, phrases, statements for practice (Appendix E).
5. Sample Trauma Narrative for role-play exercise (Appendix F).
6. “Pocket-Guide” for quick reference when taking a trauma history (Appendix G).
7. OPTIONAL: Assessment tool of beliefs, awareness, and sense of efficacy in trauma-informed care (Appendix H).

**Agenda:**

7:30am – 7:40am Review objectives of the didactic

7:40am – 7:50am Review PTSD symptoms and how they affect pregnancy

7:50am – 8:05am Identify and discuss barriers to assessing trauma history

8:05am – 8:30am Present communication template for assessing trauma history and responding to disclosures

8:30am – 8:45am Handout sample trauma-informed questions, phrases, and statements for practice

8:45am – 9:15am Practice assessing trauma history in small groups

9:15am – 9:30am Wrap-up and debrief

**In preparation for the Didactic**:

Prior to conducting this didactic and role-play exercise facilitators should ensure that they are knowledgeable about their institution’s resources for residents and other learners to address issues related to traumatic stress and mental health that may arise at any point in the context of this educational activity. It may also be helpful to prepare information regarding referrals to confidential counseling or therapy services. Additionally, facilitators should discuss with their program director(s) and be up-to-date with respect to institutional guidelines for handling/reporting of possible harassment or abuse in the context of the medical training and patient care settings as this topic (addressing trauma in the obstetric care context) may raise these issues. Also consider adding to the beginning of the didactic PowerPoint an introductory acknowledgement statement that the didactic material inherently poses a challenge to professional-personal boundaries, because trauma can affect anyone, not just patients. Consider including a reminder of confidential resources for help.

**Review Objectives of the Didactic**: (10 minutes)

Briefly introduce the topic for the didactic: Assessing trauma history of pregnant patients with histories of physical or sexual abuse and violence using trauma-informed communication. In the OB/GYN resident didactic curriculum, residents are exposed to lectures and materials that cover mental health topics and medical management of mental disorders. However, the objectives of this didactic are to achieve a deeper understanding of the effects of traumatic stress on pregnancy health and to practice empirically-supported communication skills to effectively address this problem with their patients who have experienced trauma.

Facilitators may consider administering the self-assessment tool of beliefs, awareness, and sense of efficacy in trauma-informed care prior to beginning the didactic as a pre-test and then again at the end as a post-test to demonstrate change in responses as a result of participation. The assessment tool was developed based on questions in Kassam-Adams et al. (2015) and Bruce et al. (2018).

**Review Prevalence of trauma and Impact on Pregnancy**: (10 minutes)

Follow PowerPoint slides (Appendix B). Speaker notes are offered, but may be modified based on facilitator needs. Facilitate discussion of how histories of trauma can impact pregnancy outcomes. Elicit responses that attend to physiological outcomes as well as psychological, social, and behavioral outcomes. For additional information or background reading, see articles by Blackmore et al. (2016), Geller and Stasko (2017), Seng et al. (2009), Seng et al. (2011), Yonkers et al. (2014), Yildiz et al. (2017), and ACOG Committee Opinion 777. Slides also refer to helpful resources for future use, including the Substance Use and Mental Health Services Administration’s (SAMHSA) guide on trauma-informed care (SAMHSA, 2014). In learning environments where a more in-depth review of posttraumatic stress disorder (PTSD) is desired, facilitators may use optional PowerPoint slides describing DSM-5 diagnostic criteria for PTSD defining each of the criteria A-E found at the end of the PowerPoint presentation (Appendix B).

**Identify and Discuss Barriers to Assessing trauma history in Obstetric settings**: (15 Minutes)

Present the sample case history of the pregnant patient with a trauma history (without trauma narrative). Pass out handouts for residents to reference the case/chart (Appendix C) or display on PowerPoint slides (Appendix B).

Using the following prompt, do a brainstorming exercise – write responses on blackboard if possible.

“What are some of the barriers to assessing/talking about this patient’s trauma history based on what you know from the case summary?”

Barriers and challenges to assessing trauma history should include at least a few from the following list. Ask learners to think about their experiences caring for pregnant patients, the challenges they’ve faced, and take the opportunity to normalize and validate their experiences. If no discussion ensues or learners do not generate any examples, offer the following three commonly cited barriers: Time, lack of training/knowing what to say, and fear or concern about re-traumatizing patients. The goal of this discussion is not to generate an exhaustive list or to come up with solutions for each barrier. Facilitators may consider encouraging learners to share experiences of applying certain solutions, time permitting. However, the goal should be to guide the conversation toward effective communication solutions that address the first three barriers on this list. The material in this facilitator guide is designed specifically to help overcome these barriers, i.e., to practice using empirically-supported terminology when asking about trauma and to respond empathically in the context of brief history-taking with a patient.

*Note. It may be useful to introduce the topic of providers struggling with or avoiding their own traumatic experiences as a barrier to asking patients about trauma. This topic may also emerge organically. It is important to acknowledge this as a barrier and the stigma associated with it, while also acknowledging that conducting an empirically-supported assessment of patients’ trauma histories is desirable and feasible regardless of the provider’s history. In fact, the availability of structured communication templates may be a helpful strategy for providers toward handling feelings about their own trauma experiences, to the extent that it presents a barrier to asking their patients about trauma. It may also be appropriate in this context to refer learners to resources available for addressing their own mental health, trauma, and/or secondary traumatic stress experiences provided at the beginning of the didactic.*

**Example Barriers:**

1. Time constraints
   - Concern that asking about patient’s trauma history will invariably lead to a lengthy discussion
2. Lack of training in communication about trauma
3. Fear of re-traumatizing patients
4. Environment constraints (e.g., lack of privacy, freedom from interruptions)
5. Presence of partners/family members
6. Lack of support, resources, experts to consult when patient discloses trauma history
7. Difficulties associated with providers’ own traumatic experiences

Handout #1: “Sample Chart of Pregnant Patient W/ Trauma History” (Appendix C)

Patient is a 25 year old African-American woman, G5P0131 who is 19 weeks pregnant.

*OB History:*

-- First pregnancy, age 15 ended with an elective second trimester termination.

--Second and third pregnancies, age 17 ended in spontaneous abortions at 15 weeks and 16 weeks.

--Fourth pregnancy, age 20 ended with the delivery of a preterm infant boy at 34 weeks and weighing just over 3 pounds.

--Cervical insufficiency, with fourth pregnancy.

*Medical History/Problem List:*

--Chlamydia diagnosed and treated, ages 15 and 16

--Obesity

--Asthma

--Preterm birth, age 20

--Abnormal pap, ages 19 and 21

*Surgical History:*

--Cholescystectomy, age 18

--D&C X 2, age 17 for incomplete spontaneous abortions

*Social History:*

--Marital Status: Unmarried, lives with grandmother, boyfriend (father of baby), and 4 year-old son

--Associate’s degree from City Colleges of Chicago, works part time retail

--Tobacco dependence, 1/2 PPD, quit while pregnant with her son, quit again “cold turkey” when found out was pregnant at 10 weeks

--Alcohol abuse, consumes >5 “shots” a few times per month, never during current pregnancy

--Domestic abuse, age 15 ex-boyfriend “beat her up” in high school, none with current partner

*Mental Health History:*

--Denies depression when asked. Edinburgh Postnatal Depression Scale Score = 8.

--States she has “gotten over” past abuse as long as she avoids her mother’s house where her abuser occasionally visits

**Introduce Communication Template for assessing Trauma History and Responding to Disclosures**: (25 minutes)

Remember the goals of taking a trauma-informed history:

1. Elicit, recognize, and respond to patient disclosures of past abuse, trauma, and violence.
2. Empathize and reassure the patient that she is not to blame for what happened to her.
3. Identify symptoms of emotional distress and motivate for treatment as appropriate.
4. Take steps to prevent trauma triggers by identifying trauma-informed needs/preferences for medical care.

Encourage residents they can achieve these goals by remembering the following guiding statements:

1. “It’s *not* your fault.”
2. “You *can* feel better.”
3. “Your health care should be the least scary and most comfortable experience possible.”
4. “How can I help?”

**Communication Template**

The following template (Appendix D) was developed based on the work of Paranjape, Rask, and Liebschutz (2006) and

Koss et al. (2008). Introduce the template reminding learners: Violence impacts physical and mental health; however, the majority of victims do not report their experiences. Victims often feel deep shame about these experiences. How you screen women for abuse can make them more likely to disclose and how you respond can affect your care of them during pregnancy.

**Introduce sample Trauma-informed Questions, Phrases, and Statements for Practice:** (15 minutes)

Here provide residents with specific ideas and examples to help them respond effectively and comfortably. Remember that the goal is not to elicit a detailed trauma narrative from the patient, and in fact, this can actually be contraindicated. However, residents need to be proficient in trauma-informed language, behaviorally-anchored (precise) terminology, and empathic responses to help guide the conversation from initial screening to final closure.

Distribute copies of Handout #3: “Sample Trauma-informed Questions, Phrases, and Statements.” (Appendix E). Go around the room and have each resident take turns saying each example, preferably while directly facing and speaking to one of their classmates. Briefly discuss examples that worked well and those that did not feel as comfortable to say or hear. Brainstorm alternatives and write them on the blackboard if possible.

Handout #2: “Communication Template for Assessing Trauma History and Responding to Disclosures” (Appendix D)

Pick at least one phrase from the “what to say” column for each step.

| **Step** | **Why?** | **What to say** |
| --- | --- | --- |
| 1. **Build trust** | Make the patient more likely to disclose by helping them feel comfortable. Ask others to leave because the patient may not want them to know about violence or they may be the person committing the violence. | It is important that we conduct this part of the visit with just you in order to ensure our privacy. Would you mind if your [friend, partner, family member] stepped out for a moment? |
|  |  | Many women have experienced violence. I am going to ask you some questions about violence, since these experiences can affect your health. |
|  |  | Answering questions about violence may be uncomfortable but knowing what you’ve been through helps me take better care of you. |
| 1. **Assess Physical Violence** | Make patients more likely to disclose by using specific examples of violence and unwanted sex. Patients are less likely to disclose when you use phrases like “rape” “abuse” or “domestic violence” | Have you ever been in a relationship where your partner has hit, pushed, or slapped you?  Have you ever been in a relationship where your partner threatened you with violence?  Have you ever been in a relationship where your partner has thrown, broken, or punched things?^1^ |
| 1. **Assess Sexual Violence** |  | Has anyone ever made you have intercourse, oral or anal sex against your will?  Has anyone ever touched private parts of your body, or made you touch theirs, under force or threat?  Has anyone ever taken advantage of you sexually when you were too drunk or out of it to stop it?  Have there any other situations in which another person tried to force you to have unwanted sexual contact?^2^ |
| **If the patient endorses ANY item from Step 2 or Step 3, proceed to steps 4 and 5.** | | |
| 1. **Empathize** | Validate the seriousness of abuse and the difficulty of disclosure by providing the patient with empathy. | That must have been difficult to talk about. Thank you for trusting me with this information. |
|  |  | IT IS NOT YOUR FAULT that someone hurt you. No one deserves to be treated that way. |
|  |  | You deserve to be treated with respect in all relationships. You especially deserve to feel safe and comfortable. I am concerned and would like to help. |
| 1. **Follow-up** | Provide patient with specific options for addressing trauma through medical care, mental health care, and social resources | Our clinic offers programs to help women with these experiences  [INSERT CONTACT INFO] |
|  |  | Our medical team includes psychologists who work with women who have experienced violence. They can help you cope with emotions and memories related to this experience.  [INSERT CONTACT INFO] |
|  |  | Your health care should be the least scary and most comfortable experience possible. A lot of women find aspects of their OB/GYN care uncomfortable, painful, or frightening. What parts of the visit could I make more comfortable for you? |

Handout #3: “Sample Trauma-informed Questions, Phrases, and Statements for Practice” (Appendix E)

**WHAT TO SAY**:

1. “It is important that we conduct a portion of our evaluation with just you and me in order to ensure your privacy. Your [friend, partner, family member] is most welcome to return after stepping out for this part. Do you have any questions or concerns about this part of our routine?”

2. “Violence is a major problem that has serious effects on our health. I am going to ask you some questions about some of these experiences.”

3. “These questions are very personal, so I need to make it clear that this information is confidential, meaning that it will only be shared with people who are directly involved with your medical care. Do you have any questions about this?”

4. “Unfortunately, most women have had experiences with violence or emotional mistreatment.”

5. “Have you ever been hit, slapped, or otherwise physically hurt by someone? Has this happened in your relationship with your current partner?”

6. “Has anyone ever forced you to engage in sexual activities when you didn’t want to? Has this happened in your relationship with your current partner?”

7. Have you ever felt pressured to engage in certain sexual activities that you were not comfortable with?

8. Have you ever been forced to have unwanted sex while you were unable to stop it? (i.e., if you were unconscious or too “out of it”)

9. “Thank you for trusting me with this information.”

10. “It is not your fault when someone forces you to have sex when you don’t want to. No one deserves to be treated that way.”

11. “It doesn’t matter whether the person who hurt you was a family member, friend, or significant other. It doesn’t matter where you were, if you drank too much, or were unconscious. The only person responsible for assaulting you is *the person who assaulted you.”*

12. “How are you coping with these experiences? Are you ever bothered by bad memories? Do you ever feel anxious or afraid when you think about them?”

13. “Are there situations that make you feel anxious or uncomfortable, such as medical exams?”

14. “It also sounds as if you have some uncomfortable feelings, like anxiety. This is very common when people have experienced violence, and it is a very serious problem that *can* affect the health of your pregnancy.”

15. “You deserve to be treated with respect in all relationships, and you especially deserve to feel safe and comfortable. I am concerned for you and your baby’s health and would like to help.”

16. “Part of our women’s health team includes trained psychologists who specialize in helping pregnant women who have experienced violence, or who feel worried, tense, irritable, or anxious. *You* are an important part of this team.

17. “Your health care should be the *least* scary and *most* comfortable experience possible. A lot of women find aspects of prenatal care uncomfortable, painful, or even frightening. What are the least comfortable parts of a physical exam for you?”

18. “I read in your chart that you have been seeing a counselor for therapy. What are you and the therapist working on together?”

19. “I noticed in your chart that you have a counselor but haven’t seen him/her in a while. What prompted you to stop attending sessions?”

20. “What strategies has your counselor given you to help with emotional difficulties, like anxiety or depression?”

**WHAT NOT TO SAY**:

1. “You haven’t had any issues with trauma, right?” (Leading question)

2. “Any trauma or abuse?” (Not a full question; semi-leading; vague terminology)

3. “Have you ever been raped?” (Vague terminology; open to various interpretations)

4. “Has anyone ever molested you?” (Vague terminology; open to various interpretations)

5. “Has anyone ever touched you inappropriately?” (Vague terminology; open to various interpretations)

6. “Have you experienced domestic violence?” (Vague terminology; open to various interpretations)

7. “Any issues with abuse you want to talk about?” (See #2; patient may not want to talk about it and still appreciate the opportunity to disclose)

8. “I know how you feel.” (Generally, self-disclosure is best avoided as providers don’t necessarily “know” how a patient is feeling.)

9. “Why didn’t you report it/tell someone?” (Victim-blaming)

10. “Were you drunk/intoxicated? What were you doing/wearing at the time?” (Victim-blaming; It is not provider’s responsibility to determine veracity of the patient’s disclosure).

**REMEMBER: SCREENING FOR TRAUMA REQUIRES A COMBINATION OF OPEN- AND CLOSED-ENDED QUESTIONS TO GATHER ACCURATE INFORMATION FROM THE PATIENT. AVOID LEADING QUESTIONS AND NONSPECIFIC PHRASES. THESE ARE LEAST LIKELY TO ELICIT ACCURATE RESPONSES.**

**Practice Taking a Trauma History in Small Groups**: (30 Minutes)

Ask the group to divide into pairs or groups of three. If there are multiple facilitators, depending on the size of the group, one facilitator can pretend to be the patient described earlier whose trauma history is detailed in the Sample Trauma Narrative for Role-Play Exercise tool (Appendix F). Alternatively, the residents can take turns pretending to be the patient while their partner interviews them and then switch places so the first interviewer becomes the “patient.” Instruct the facilitator or learner playing the patient that they should not expect to give all the responses from the narrative. During the interview, encourage residents to provide each other with constructive feedback. Move between groups/pairs to observe and provide your feedback as well.

Facilitators can give the person role-playing the “patient” a few moments to review the narrative. This is a fictional trauma narrative of the patient we introduced earlier. The goal is NOT to detail all the information that you need to elicit from a trauma history but rather to be able to identify key pieces of information that might affect the patient’s obstetric care and to think of ways you would respond if a patient did disclose any of this information in their care with you. Hand out “pocket-guides” for interviewers to use during their practice. **Role-play instructions**:

- *We would like for you to role-play an interview of a patient in group “round-robin” format.*
- *In particular, we want you to do a trauma screening as you would for any new patient.*
- *We have provided you with a pocket guide that has sample phrases for five domains – we want you, as a group, to cover each of these areas.*
- *When it’s your turn, you can either 1) ask a question or 2) make an empathic/reflective statement – whichever feels most appropriate.*
- *Establish an order.*
- *After the interview, invite the group to give one another feedback.*
- *Add your own feedback.*

After the role-play/practice, ask learners to reflect on and discuss the information that you think would be relevant to the patient’s obstetric care and how you would respond if the patient disclosed any of the information to you.

Providing Feedback to Learners:

Before concluding the module, provide constructive feedback to learners both on their reflections and on the communication observed in the role-play exercise. There are several examples that offer key learning opportunities. First, recognize when a learner veers “off script” as this may indicate a discomfort or lack of confidence with the material or, conversely, it may reflect exceptional comfort and ability to flexibly apply various terminology to different patient care scenarios while still achieving the communication objectives. Second, recognize long pauses or stumbling over words is an indicator that more practice is needed and possibly an assessment of other barriers. Third, recognize when the patient alludes to a potentially traumatic event or abusive situation and the learner does not follow up, as this can indicate avoidance. Finally, recognize if the learner uses responses such as “I’m sorry to hear that” or “I’m sorry that happened to you” too frequently. Encourage rephrasing to a more empathic reflection, such as “It sounds like that was a terrifying experience. How are you coping now?” or “It sounds like this is upsetting to think about. Have you ever talked to anyone about it/would you be interested in talking to someone about it?”

*Some examples of specific feedback include the following suggestions:*

1. “Setting up” the question – It is appropriate to include a “set-up” (Handout #3, Items 1-4) but take care not to “apologize” for asking the question as this can reinforce stigma.

2. Avoid leading or half-questions that are not really questions, e.g., “Any/No trauma or abuse in your life?”

3. Avoid vague terminology that could be interpreted to mean multiple things, e.g., “Have you been raped/molested?”

4. When in doubt, stick to the script, especially if you find yourself struggling to “find the right words.”

5. If a word or phrase makes you uncomfortable or is difficult to say out loud, consider practicing saying it over and over (alone or practice with a peer).

6. Be VERY selective in using “alternatives” to phrases and terms described in the script as these can often contain vague language that is open to interpretation (e.g., rape, molestation, trauma).

7. Avoid victim-blaming language. Be aware that certain questions (e.g., Were you intoxicated? Why didn’t you report it?) suggest blaming.

8. Avoid asking questions about the details of the event as these are not necessary to assess an accurate history. The provider’s task is not to determine veracity. Eliciting specific trauma details can trigger or exacerbate trauma symptoms.

**References**

1. Kassam-Adams N, Rzucidlo S, Campbell M, Good G, Bonifacio E, Slouf K, Schneider S, McKenna C, Hanson CA, Grather D. Nurses' views and current practice of trauma-informed pediatric nursing care. J Pediatr Nurs. 2015;30(3):478-84.
2. Bruce MM, Kassam-Adams N, Rogers M, Anderson KM, Sluys KP, Richmond TS. Trauma providers' knowledge, views, and practice of trauma-informed care. J Trauma Nurs 2018;25(2):131-8.
3. Blackmore ER, Putnam FW, Pressman EK, Rubinow DR, Putnam KT, Matthieu MM, Gilchrist MA, Jones I, O'Connor TG. The effects of trauma history and prenatal affective symptoms on obstetric outcomes. J Trauma Stress 2016;29(3):245-52.
4. Geller PA, Stasko EC. Effect of previous posttraumatic stress in the perinatal period. JOGNN J Obstet Gynecol Neonatal Nurs 2017;46(6):912-22.
5. Seng JS, Low LK, Sperlich M, Ronis DL, Liberzon I. Prevalence, trauma history, and risk for posttraumatic stress disorder among nulliparous women in maternity care. *Obstet Gynecol*. 2009;114(4):839–847. doi:10.1097/AOG.0b013e3181b8f8a2
6. Seng JS, Low LK, Sperlich M, Ronis DL, Liberzon I. Post-traumatic stress disorder, child abuse history, birthweight and gestational age: a prospective cohort study. *BJOG*. 2011;118(11):1329–1339. doi:10.1111/j.1471-0528.2011.03071.x
7. Yonkers KA, Smith MV, Forray A, et al. Pregnant Women With Posttraumatic Stress Disorder and Risk of Preterm Birth. *JAMA Psychiatry.* 2014;71(8):897–904.
8. Yildiz PD, Ayers S, Phillips L. The prevalence of posttraumatic stress disorder in pregnancy and after birth: A systematic review and meta-analysis. J Affective Disord 2017;208:634-45.
9. Sexual Assault. ACOG Committee Opinion No. 777. American College of Obstetricians and Gynecologists. Obstet Gynecol 2019; 133:e296-302.
10. Substance Abuse and Mental Health Services Administration. SAMHSA’s Concept of Trauma and Guidance for a Trauma-Informed Approach. HHS Publication No. (SMA) 14-4884. Rockville, MD: Substance Abuse and Mental Health Services Administration, 2014
11. Koss, M. P., Abbey, A., Campbell, R., Cook, S., Norris, J., Testa, M., . . . White, J. (2008). Revising the SES: A collaborative process to improve assessment of sexual aggression and victimization (psychology of women quarterly (2007) 31, (357-370).*Psychology of Women Quarterly, 32*(4), 493. doi:10.1111/j.1471-6402.2008.00468.
12. Paranjape, A., Rask, K., & Liebschutz, J. (2006). Utility of STaT for the identification of recent intimate partner violence.*Journal of the National Medical Association, 98*(10), 1663-1669.
