# Supplementary material for: Assessing Trauma History in Pregnant Patients: A Didactic Module and Role-Play for Obstetrics and Gynecology Residents
Source: MedEdPORTAL. 2020 Jul 20;16:10925. doi: 10.15766/mep_2374-8265.10925 (PMC7373354; doi:10.15766/mep_2374-8265.10925)
Supplement: Supplementary file 1 — Didactic Facilitator Guide.docxPowerPoint Slides.pptxHandout 1 Sample Chart of Pregnant Patient With PTSD.docxHandout 2 Communication Template.docxHandout 3 Sample Trauma-Informed Practice.docxHandout 4 Sample Trauma Narrative for Role-Play.docxPocket Guide for Trauma History Screening.pdfAssessment Tool.docx [file mep_2374-8265.10925-s001.zip › B. PowerPoint Slides.pptx]

## Slide 1
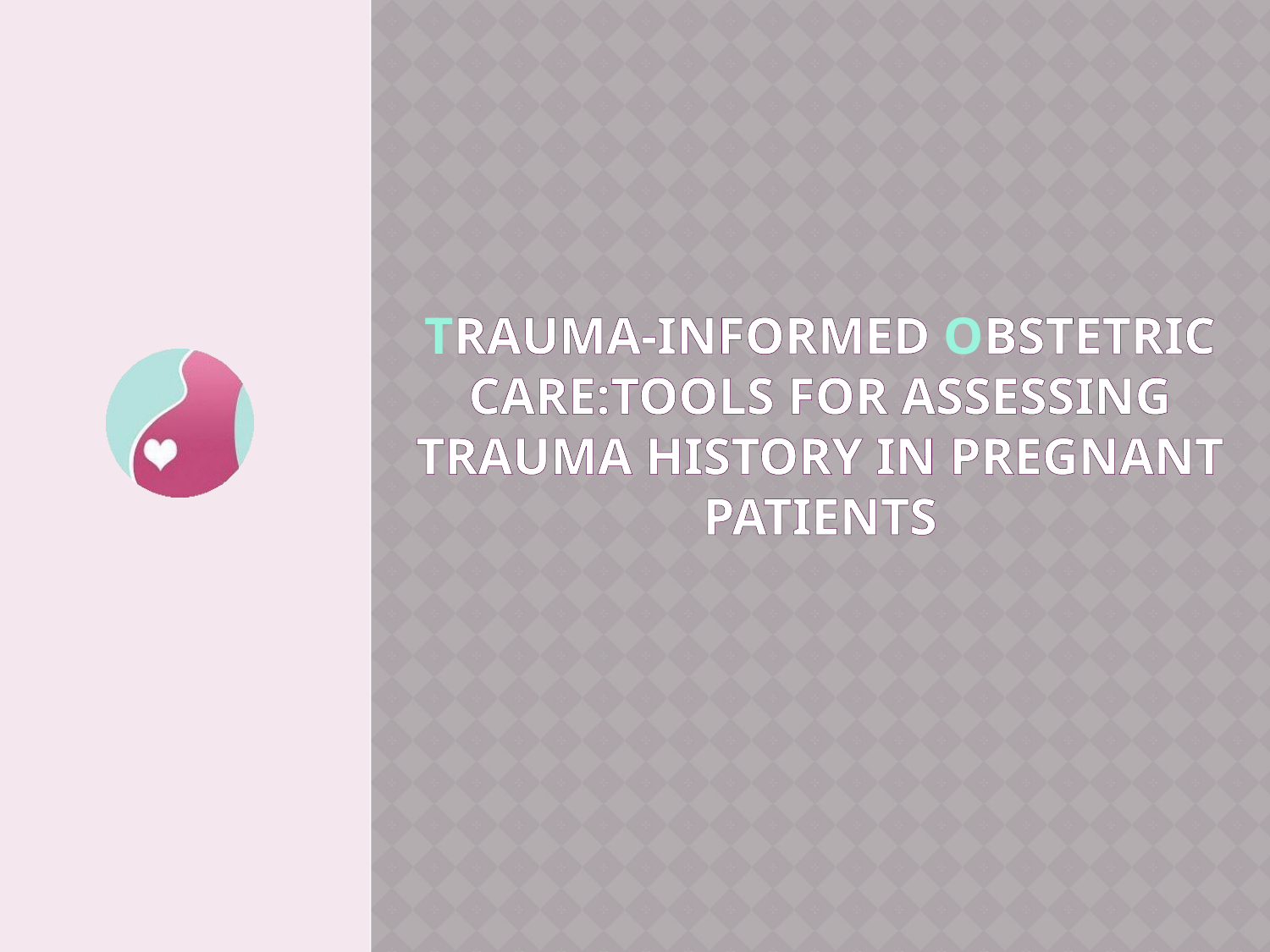

Trauma-Informed Obstetric Care:Tools for assessing trauma history in Pregnant PAtients

## Slide 2
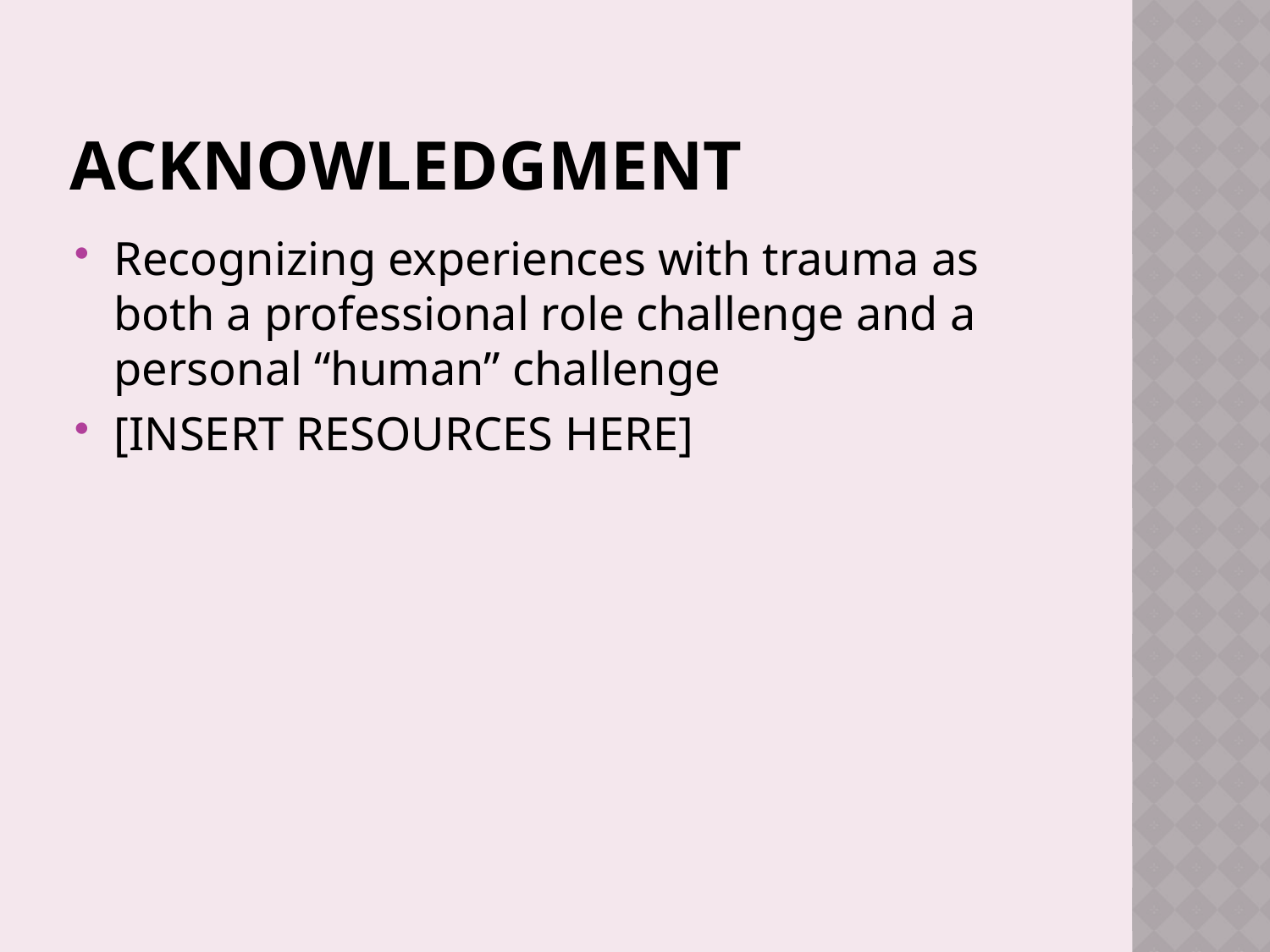

# Acknowledgment
Recognizing experiences with trauma as both a professional role challenge and a personal “human” challenge
[INSERT RESOURCES HERE]

## Slide 3
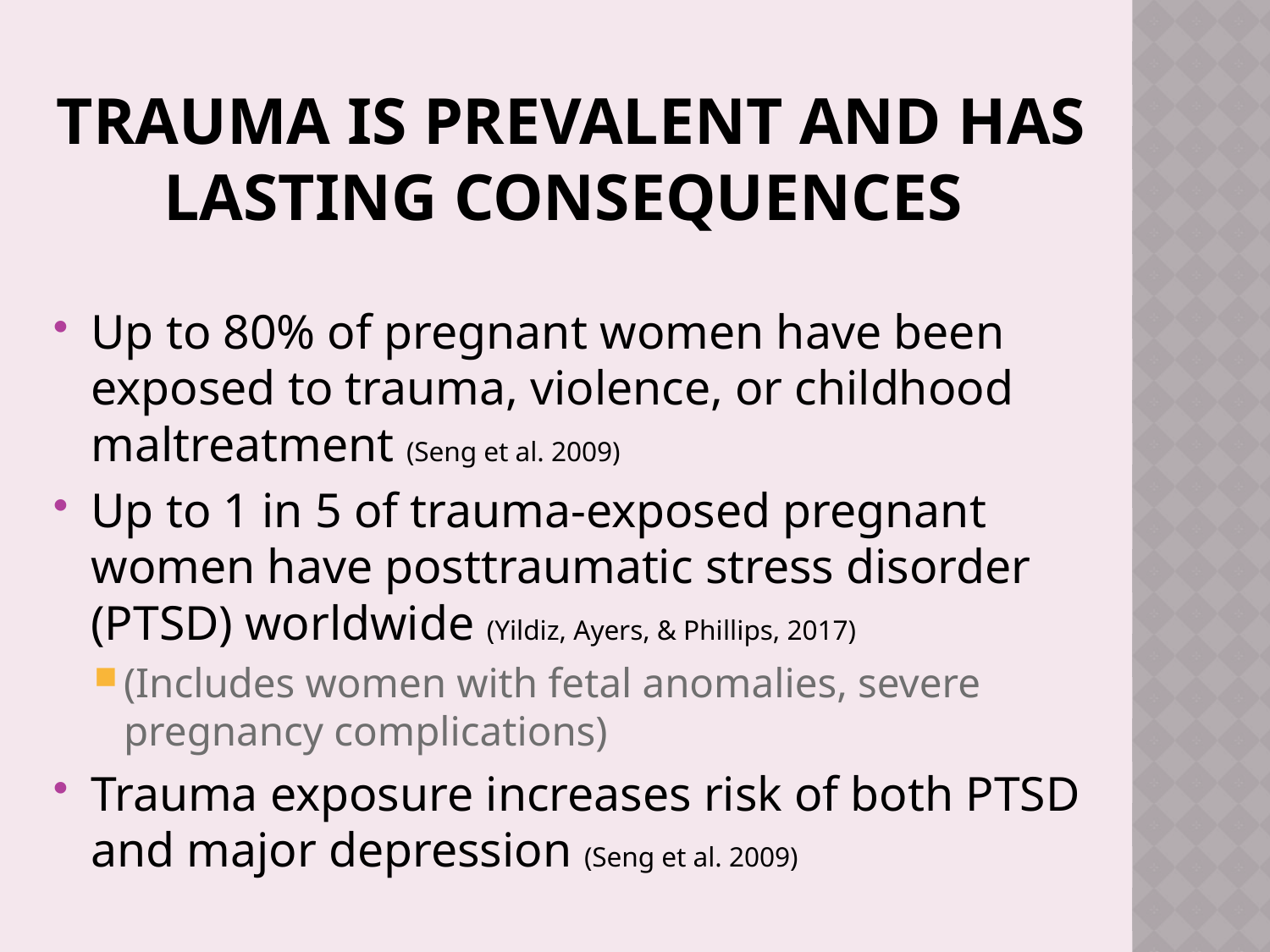

# Trauma is Prevalent and Has Lasting Consequences
Up to 80% of pregnant women have been exposed to trauma, violence, or childhood maltreatment (Seng et al. 2009)
Up to 1 in 5 of trauma-exposed pregnant women have posttraumatic stress disorder (PTSD) worldwide (Yildiz, Ayers, & Phillips, 2017)
(Includes women with fetal anomalies, severe pregnancy complications)
Trauma exposure increases risk of both PTSD and major depression (Seng et al. 2009)

## Slide 4
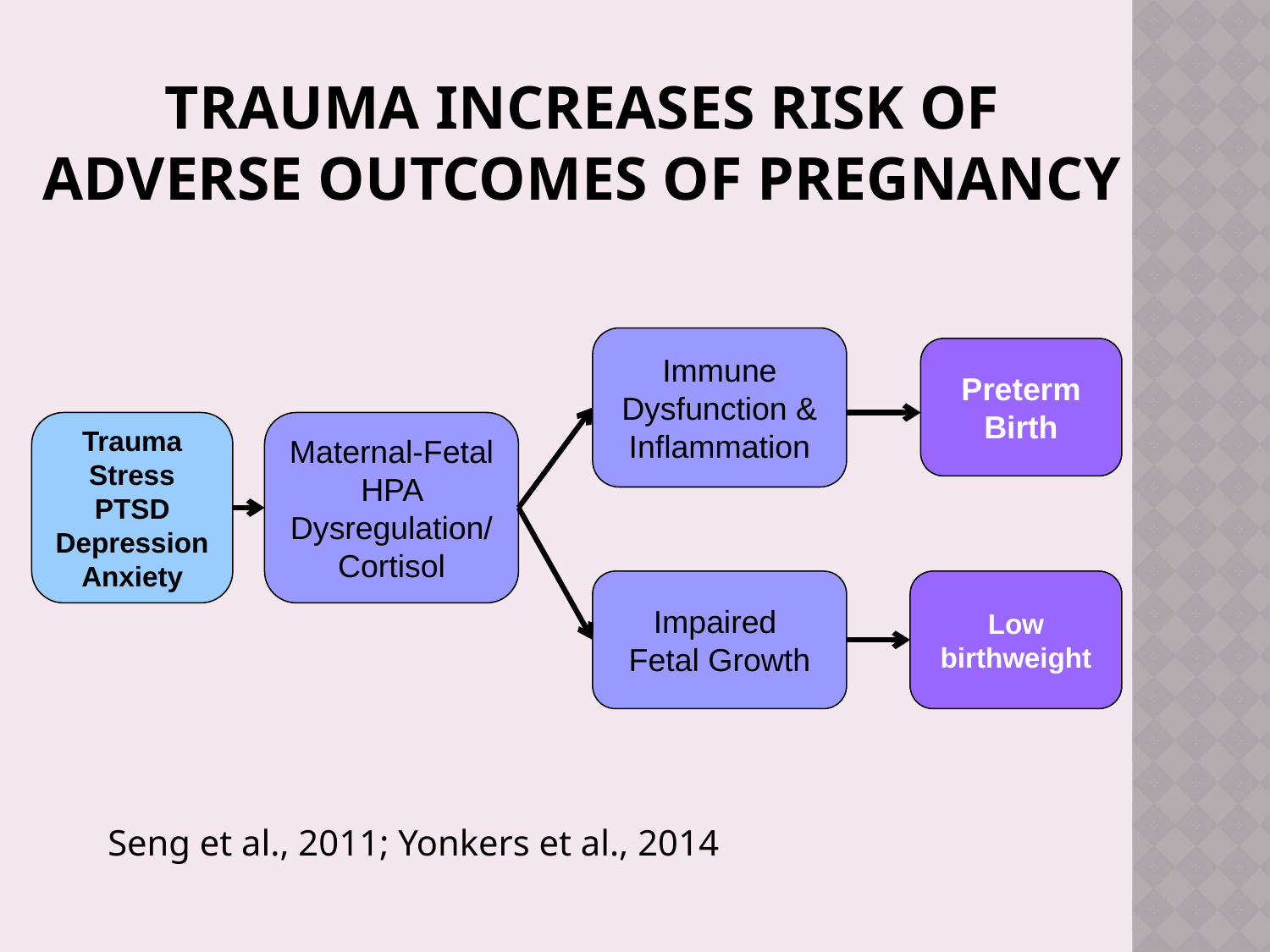

# Trauma IncreaseS risk of Adverse outcomes of pregnancy
Immune Dysfunction & Inflammation
Preterm Birth
Trauma Stress
PTSD
Depression
Anxiety
Maternal-Fetal HPA Dysregulation/Cortisol
Impaired
Fetal Growth
Low birthweight
Seng et al., 2011; Yonkers et al., 2014

## Slide 5
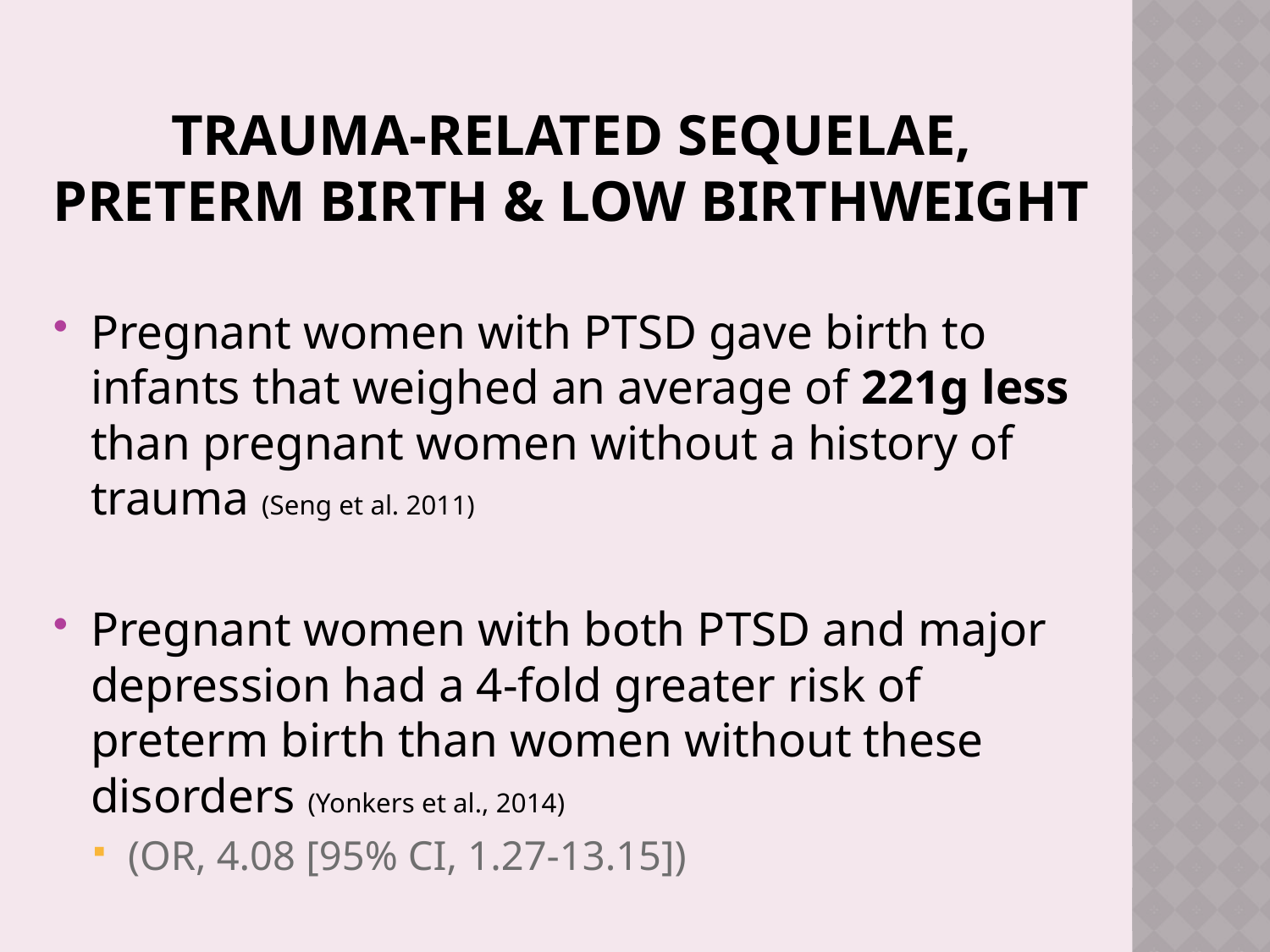

# Trauma-Related sequelae, preterm birth & low Birthweight
Pregnant women with PTSD gave birth to infants that weighed an average of 221g less than pregnant women without a history of trauma (Seng et al. 2011)
Pregnant women with both PTSD and major depression had a 4-fold greater risk of preterm birth than women without these disorders (Yonkers et al., 2014)
 (OR, 4.08 [95% CI, 1.27-13.15])

## Slide 6
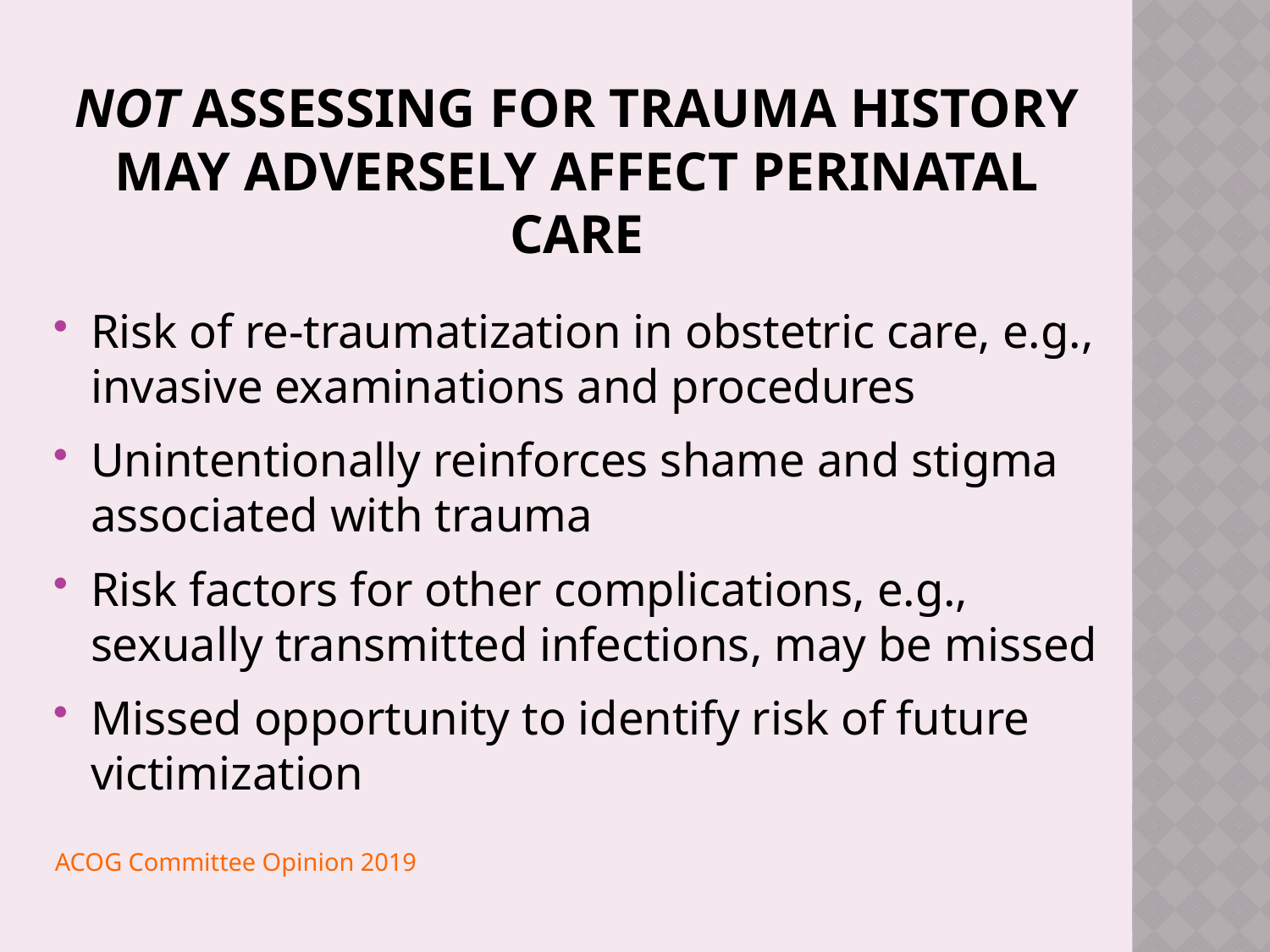

# NOT assessing for trauma history may adversely affect perinatal care
Risk of re-traumatization in obstetric care, e.g., invasive examinations and procedures
Unintentionally reinforces shame and stigma associated with trauma
Risk factors for other complications, e.g., sexually transmitted infections, may be missed
Missed opportunity to identify risk of future victimization
ACOG Committee Opinion 2019

## Slide 7
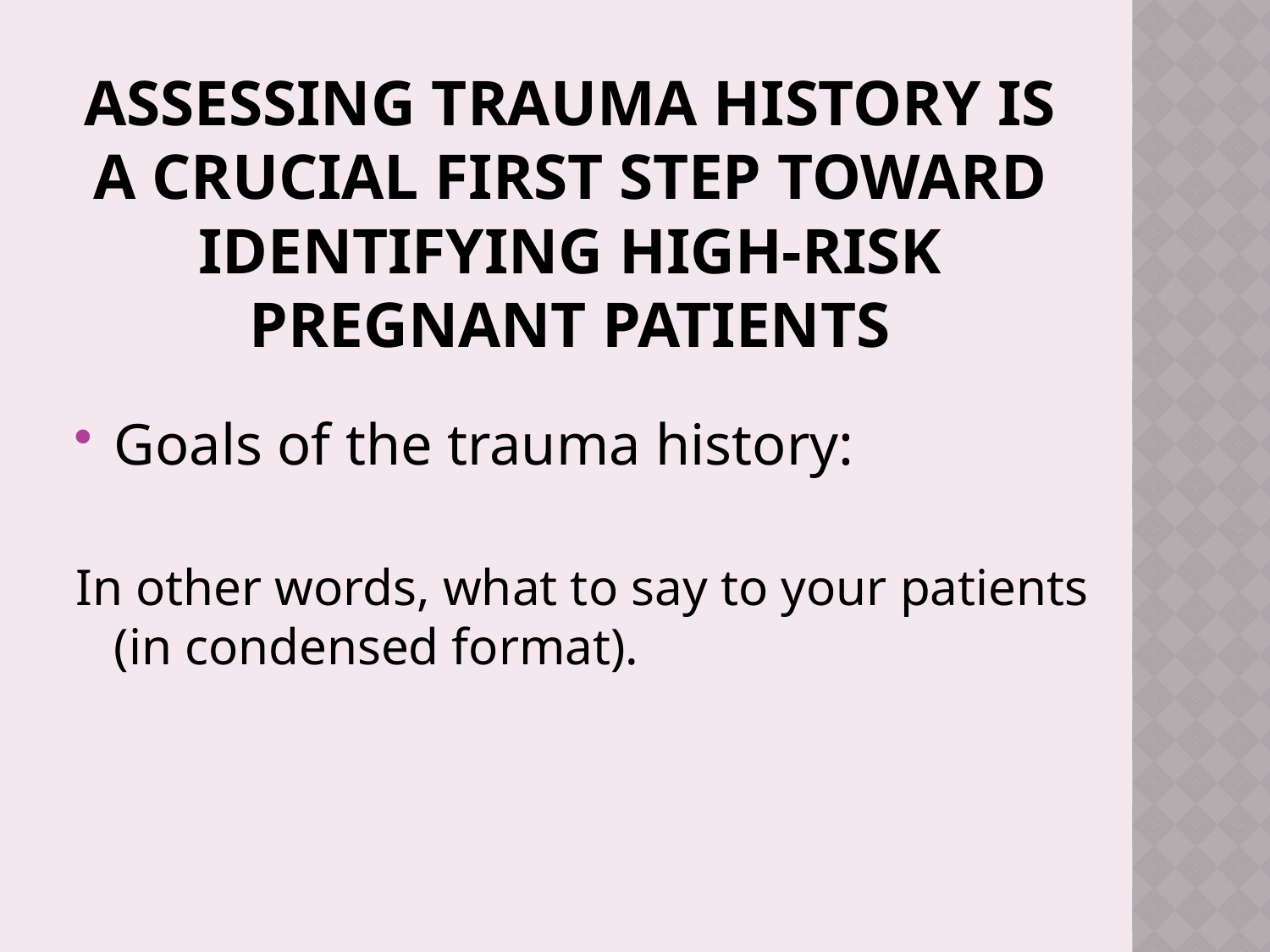

# Assessing trauma history is a crucial first step toward identifying High-Risk pregnant patients
Goals of the trauma history:
In other words, what to say to your patients (in condensed format).

## Slide 8
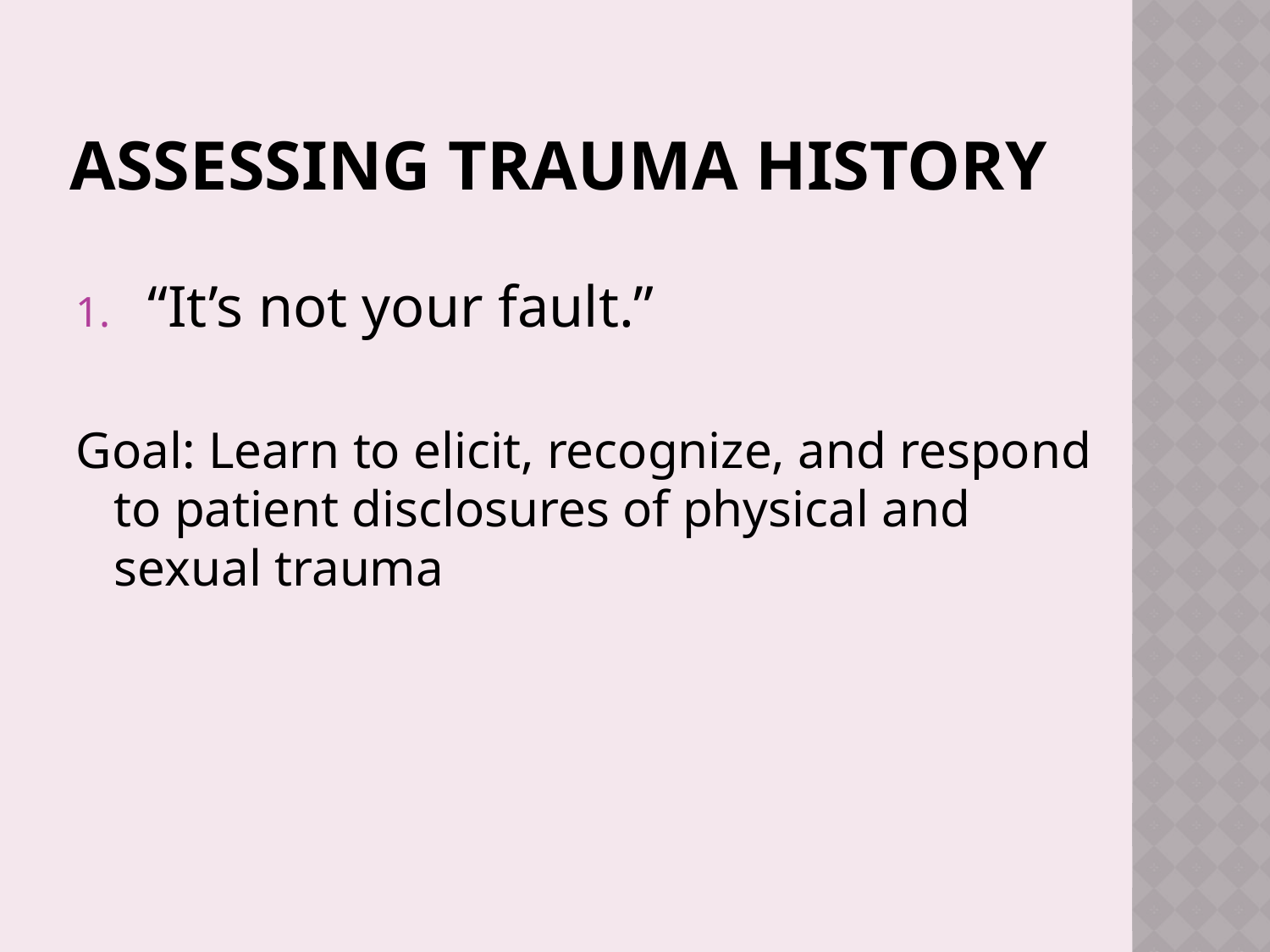

# Assessing trauma history
“It’s not your fault.”
Goal: Learn to elicit, recognize, and respond to patient disclosures of physical and sexual trauma

## Slide 9
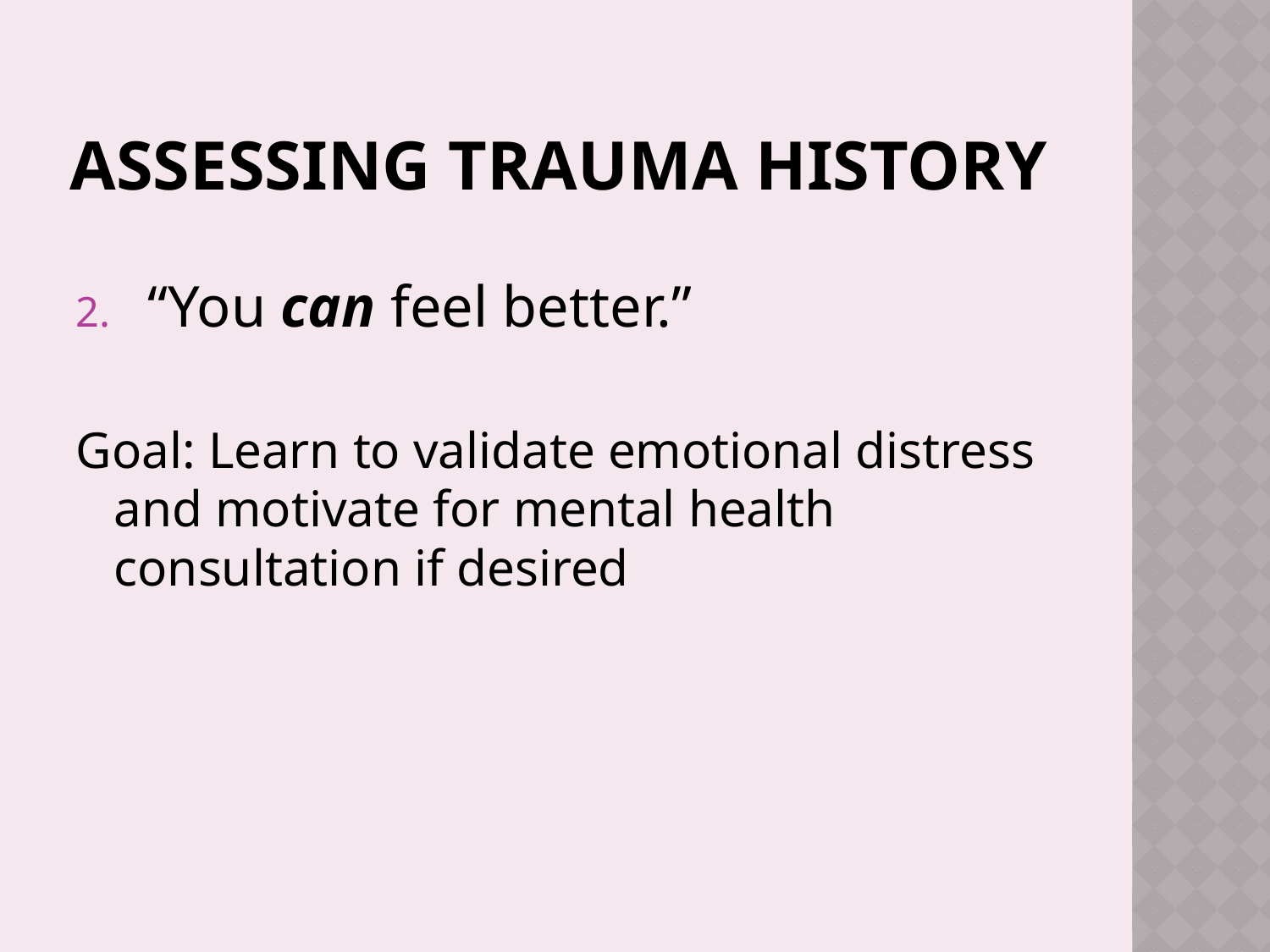

# Assessing trauma history
“You can feel better.”
Goal: Learn to validate emotional distress and motivate for mental health consultation if desired

## Slide 10
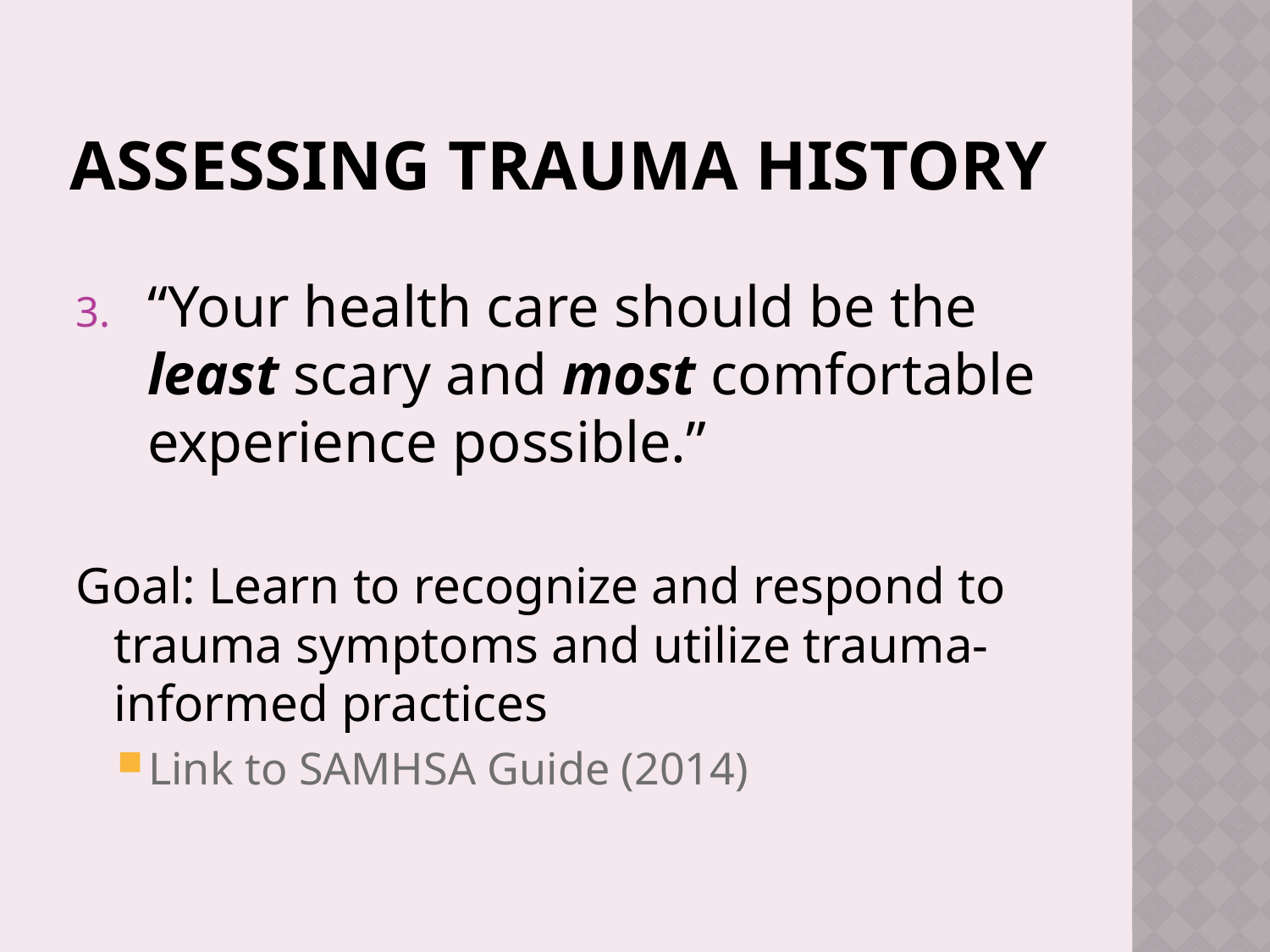

# Assessing trauma history
“Your health care should be the least scary and most comfortable experience possible.”
Goal: Learn to recognize and respond to trauma symptoms and utilize trauma-informed practices
Link to SAMHSA Guide (2014)

## Slide 11
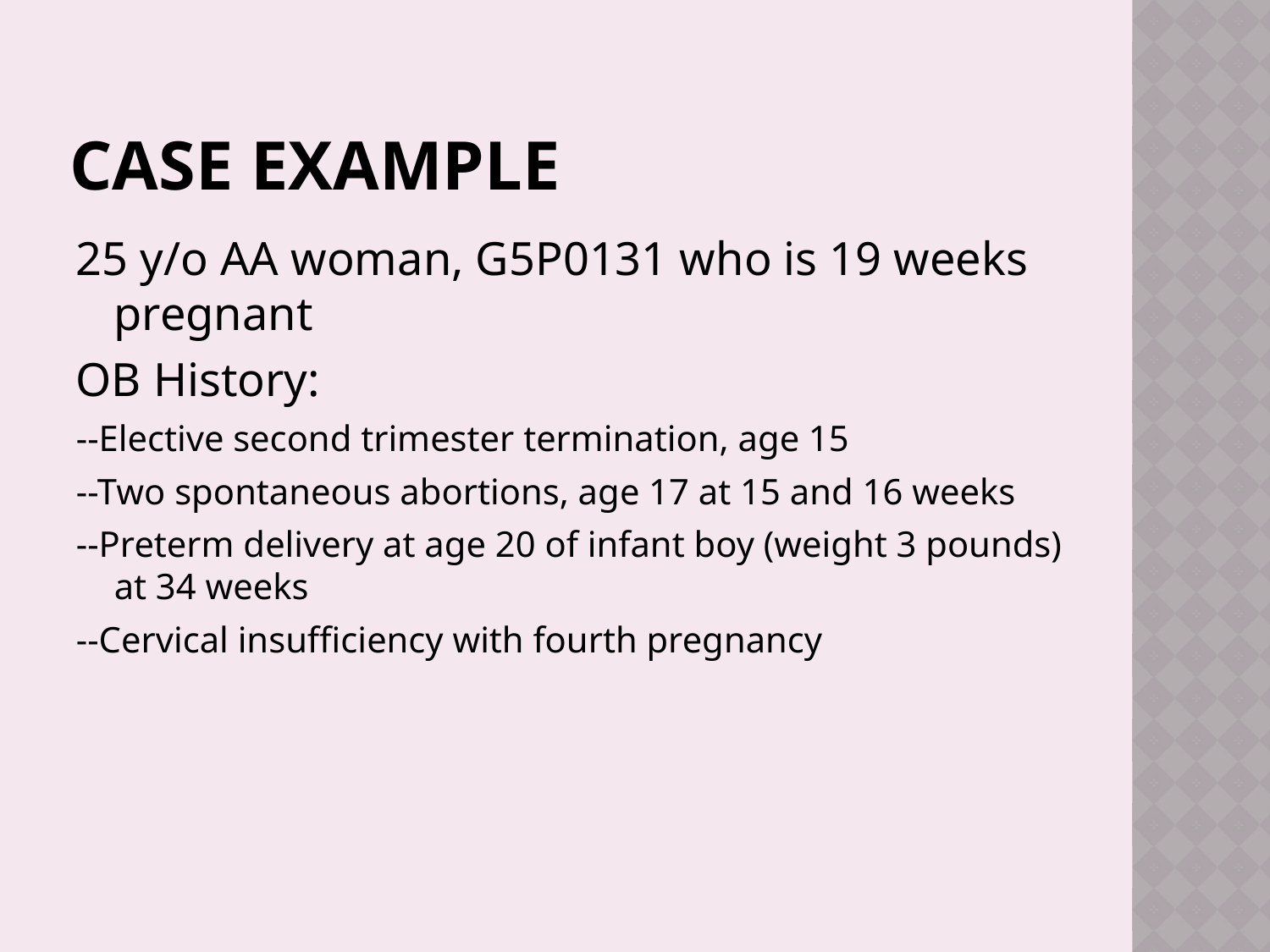

# CASE Example
25 y/o AA woman, G5P0131 who is 19 weeks pregnant
OB History:
--Elective second trimester termination, age 15
--Two spontaneous abortions, age 17 at 15 and 16 weeks
--Preterm delivery at age 20 of infant boy (weight 3 pounds) at 34 weeks
--Cervical insufficiency with fourth pregnancy

## Slide 12
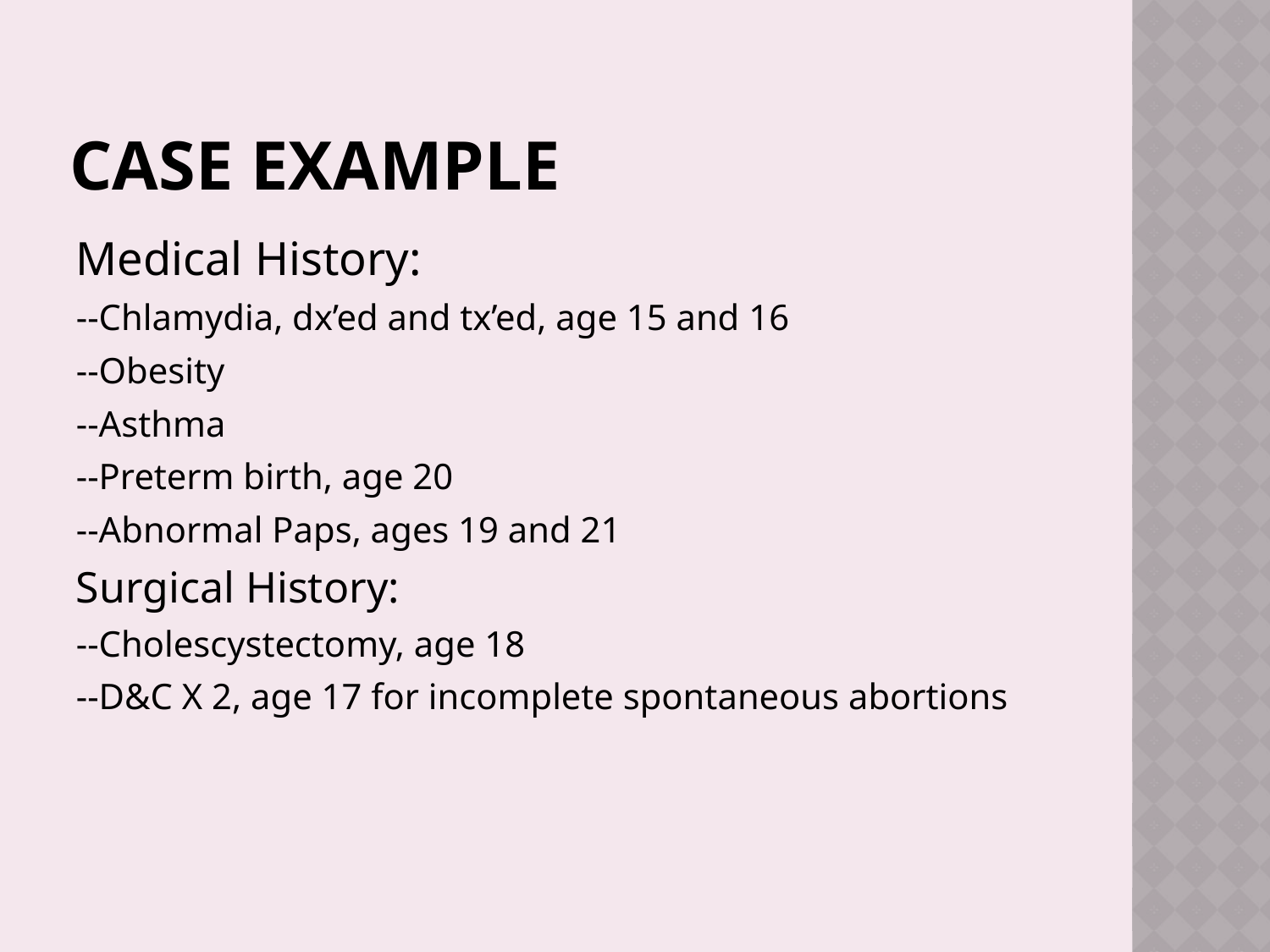

# CASE Example
Medical History:
--Chlamydia, dx’ed and tx’ed, age 15 and 16
--Obesity
--Asthma
--Preterm birth, age 20
--Abnormal Paps, ages 19 and 21
Surgical History:
--Cholescystectomy, age 18
--D&C X 2, age 17 for incomplete spontaneous abortions

## Slide 13
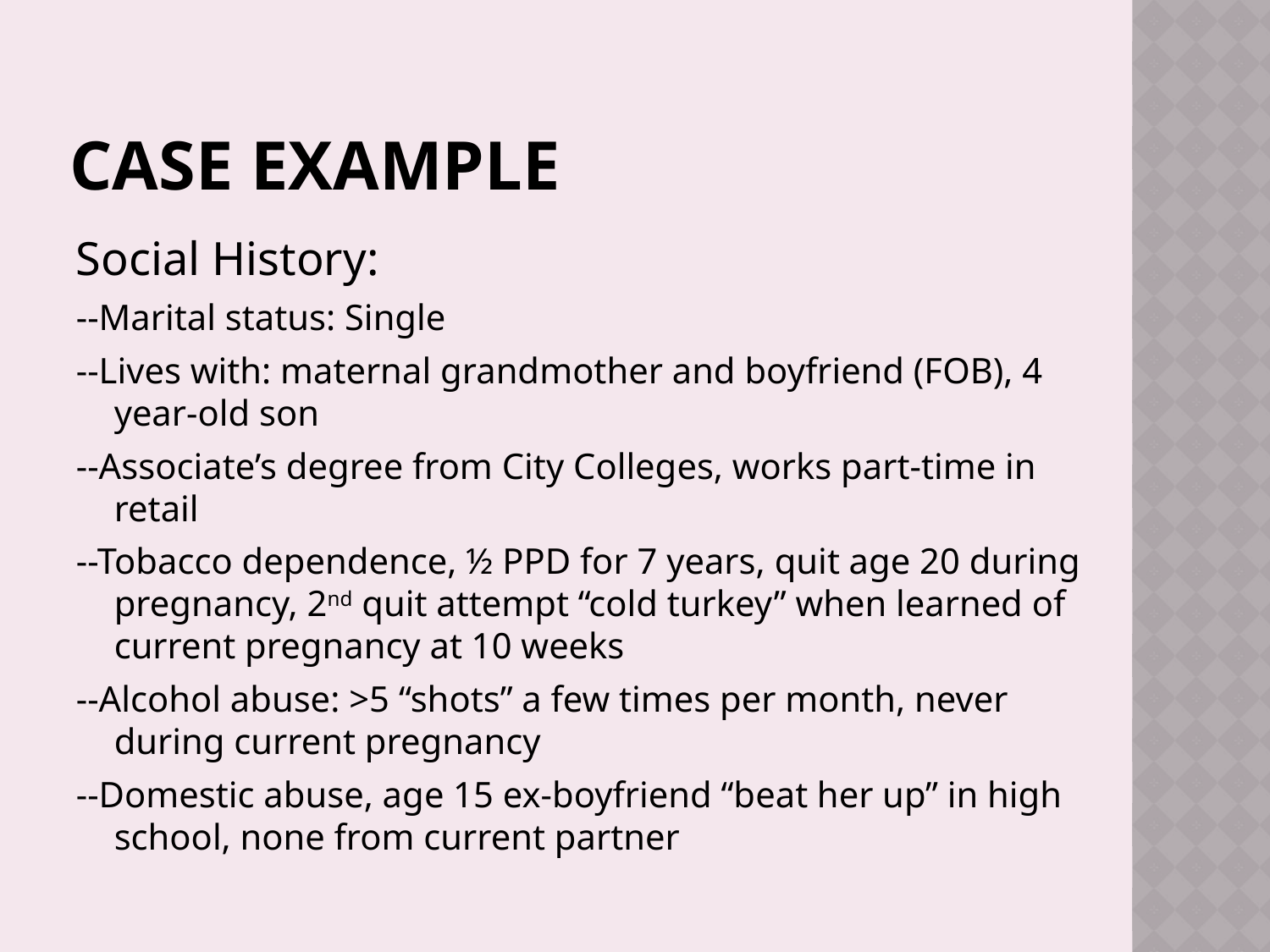

# CASE Example
Social History:
--Marital status: Single
--Lives with: maternal grandmother and boyfriend (FOB), 4 year-old son
--Associate’s degree from City Colleges, works part-time in retail
--Tobacco dependence, ½ PPD for 7 years, quit age 20 during pregnancy, 2nd quit attempt “cold turkey” when learned of current pregnancy at 10 weeks
--Alcohol abuse: >5 “shots” a few times per month, never during current pregnancy
--Domestic abuse, age 15 ex-boyfriend “beat her up” in high school, none from current partner

## Slide 14
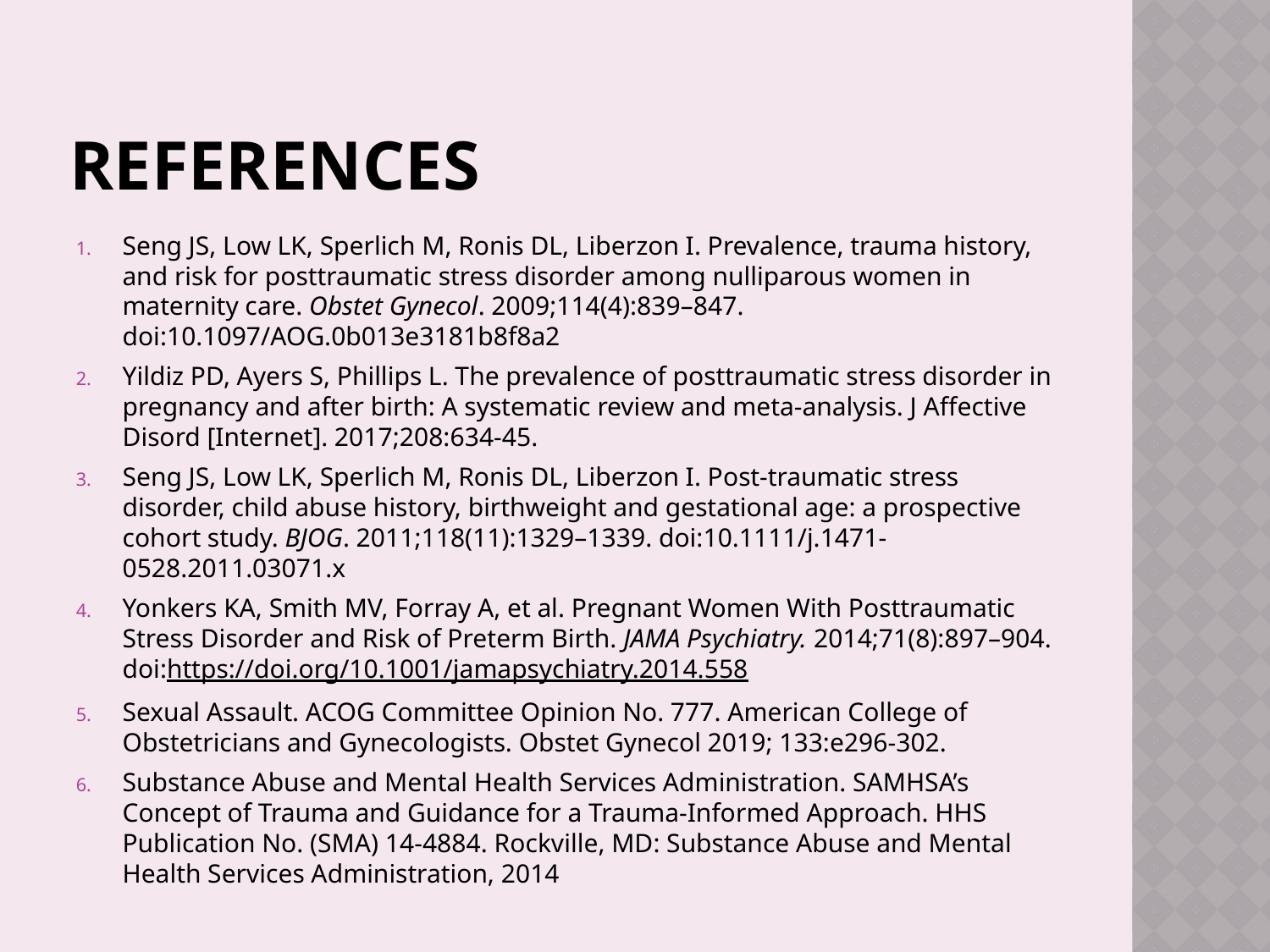

# References
Seng JS, Low LK, Sperlich M, Ronis DL, Liberzon I. Prevalence, trauma history, and risk for posttraumatic stress disorder among nulliparous women in maternity care. Obstet Gynecol. 2009;114(4):839–847. doi:10.1097/AOG.0b013e3181b8f8a2
Yildiz PD, Ayers S, Phillips L. The prevalence of posttraumatic stress disorder in pregnancy and after birth: A systematic review and meta-analysis. J Affective Disord [Internet]. 2017;208:634-45.
Seng JS, Low LK, Sperlich M, Ronis DL, Liberzon I. Post-traumatic stress disorder, child abuse history, birthweight and gestational age: a prospective cohort study. BJOG. 2011;118(11):1329–1339. doi:10.1111/j.1471-0528.2011.03071.x
Yonkers KA, Smith MV, Forray A, et al. Pregnant Women With Posttraumatic Stress Disorder and Risk of Preterm Birth. JAMA Psychiatry. 2014;71(8):897–904. doi:https://doi.org/10.1001/jamapsychiatry.2014.558
Sexual Assault. ACOG Committee Opinion No. 777. American College of Obstetricians and Gynecologists. Obstet Gynecol 2019; 133:e296-302.
Substance Abuse and Mental Health Services Administration. SAMHSA’s Concept of Trauma and Guidance for a Trauma-Informed Approach. HHS Publication No. (SMA) 14-4884. Rockville, MD: Substance Abuse and Mental Health Services Administration, 2014

## Slide 15
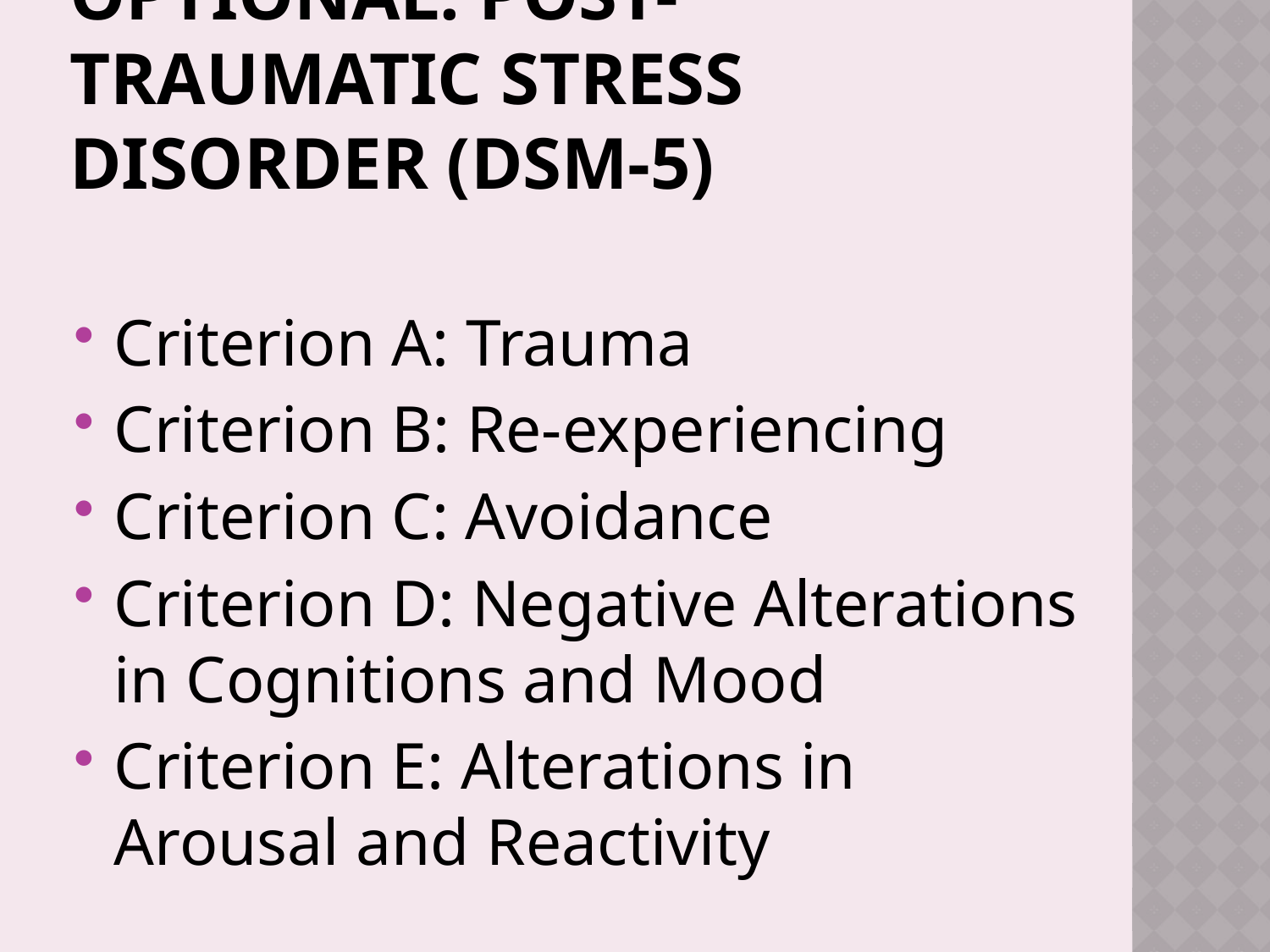

# OPTIONAL: Post-traumatic stress disorder (DSM-5)
Criterion A: Trauma
Criterion B: Re-experiencing
Criterion C: Avoidance
Criterion D: Negative Alterations in Cognitions and Mood
Criterion E: Alterations in Arousal and Reactivity

## Slide 16
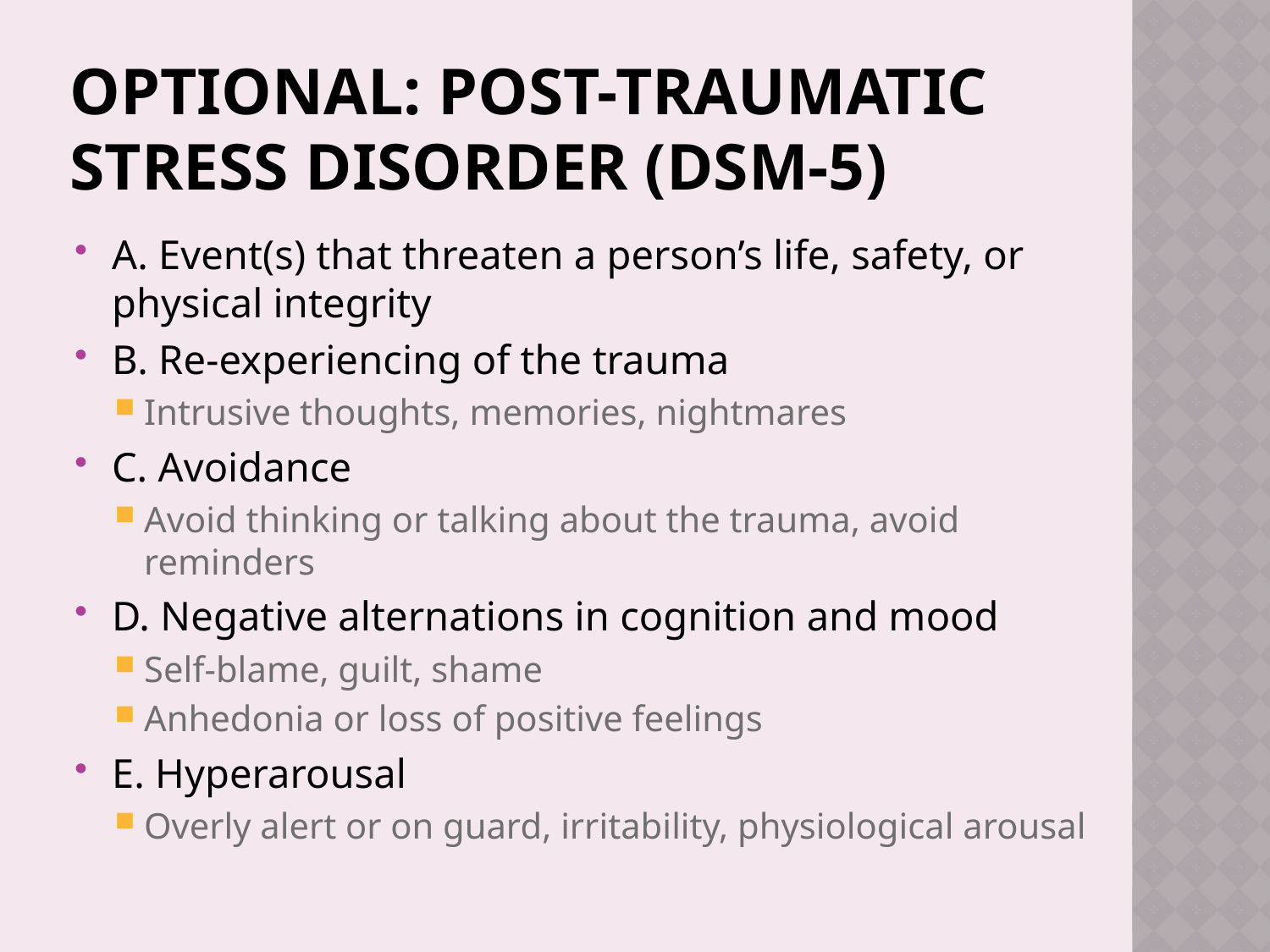

# Optional: Post-traumatic stress disorder (DSM-5)
A. Event(s) that threaten a person’s life, safety, or physical integrity
B. Re-experiencing of the trauma
Intrusive thoughts, memories, nightmares
C. Avoidance
Avoid thinking or talking about the trauma, avoid reminders
D. Negative alternations in cognition and mood
Self-blame, guilt, shame
Anhedonia or loss of positive feelings
E. Hyperarousal
Overly alert or on guard, irritability, physiological arousal
