# Supplementary material for: Assessing Trauma History in Pregnant Patients: A Didactic Module and Role-Play for Obstetrics and Gynecology Residents
Source: MedEdPORTAL. 2020 Jul 20;16:10925. doi: 10.15766/mep_2374-8265.10925 (PMC7373354; doi:10.15766/mep_2374-8265.10925)
Supplement: Supplementary file 1 — Didactic Facilitator Guide.docxPowerPoint Slides.pptxHandout 1 Sample Chart of Pregnant Patient With PTSD.docxHandout 2 Communication Template.docxHandout 3 Sample Trauma-Informed Practice.docxHandout 4 Sample Trauma Narrative for Role-Play.docxPocket Guide for Trauma History Screening.pdfAssessment Tool.docx [file mep_2374-8265.10925-s001.zip › C. Handout 1 Sample Chart of Pregnant Patient with PTSD.docx]

Handout #1: “Sample Chart of Pregnant Patient W/ PTSD”

**Initial Office Visit – Pregnancy**

**CHIEF COMPLAINT**:

| Pregnancy. |
| --- |

**HISTORY OF PRESENT ILLNESS**:

Patient is a 25-yo African American woman who presents for her first prenatal appointment; she believes that she is about 19 weeks pregnant. Pt is G5P0131. Her first pregnancy, at age 15, ended with an elective second-trimester termination. Her second and third pregnancies, at age 17, ended in spontaneous abortions at 15 and 16 weeks, respectively. Her fourth pregnancy, at age 20, ended with the delivery of a preterm infant boy at 34 weeks and weighing 3 lbs 1 oz. Cervical insufficiency with fourth pregnancy.

| **PROBLEM LIST / PAST MEDICAL HISTORY** |
| --- |
| - Chlamydia diagnosed and treated, at ages 15 and 16 - Obesity - Asthma - Preterm birth, age 20 - Abnormal pap, ages 19 and 21 |
| **MEDICATIONS** |
| - None reported |
| **ALLERGIES** |
| - PCN (penicillin) – HIVES |
| **PAST SURGICAL HISTORY** |
| - Cholescystectomy, age18 - D&C X2, age 17, for incomplete spontaneous abortions |

**SOCIAL AND FAMILY HISTORY**:

- Unmarried, lives with grandmother, boyfriend (father of baby), and 4-yo son
- Associate’s degree from City Colleges of Chicago, works part-time retail
- Tobacco dependence, ½ PPD, quit 5 years ago while pregnant w/ her son, quit again “cold turkey” when found out she was pregnant at 10 wks
- Alcohol abuse, consumes >5 “shots” a few times per month, never during current pregnancy
- Domestic abuse, age 15 ex-boyfriend “beat her up” in high school, none with current partner

**MENTAL HEALTH HISTORY**

- Edinburgh Postnatal Depression Scale Score = 8. (Denies depression when asked).
- Previous visit notes: Patient states she has “gotten over” past abuse as long as she avoids her mother’s house where her abuser occasionally visits
