# Supplementary material for: Assessing Trauma History in Pregnant Patients: A Didactic Module and Role-Play for Obstetrics and Gynecology Residents
Source: MedEdPORTAL. 2020 Jul 20;16:10925. doi: 10.15766/mep_2374-8265.10925 (PMC7373354; doi:10.15766/mep_2374-8265.10925)
Supplement: Supplementary file 1 — Didactic Facilitator Guide.docxPowerPoint Slides.pptxHandout 1 Sample Chart of Pregnant Patient With PTSD.docxHandout 2 Communication Template.docxHandout 3 Sample Trauma-Informed Practice.docxHandout 4 Sample Trauma Narrative for Role-Play.docxPocket Guide for Trauma History Screening.pdfAssessment Tool.docx [file mep_2374-8265.10925-s001.zip › D. Handout 2 Communication Template.docx]

Handout #2: “Communication Template for Assessing Trauma History”

Pick at least one phrase from the “what to say” column for each step.

| **Step** | **Why?** | **What to say** |
| --- | --- | --- |
| 1. **Build trust** | Make the patient more likely to disclose by helping them feel comfortable. Ask others to leave because the patient may not want them to know about violence or they may be the person committing the violence. | It is important that we conduct this part of the visit with just you in order to ensure our privacy. Would you mind if your [friend, partner, family member] stepped out for a moment? |
|  |  | Many women have experienced violence. I am going to ask you some questions about violence, since these experiences can affect your health. |
|  |  | Answering questions about violence may be uncomfortable but knowing what you’ve been through helps me take better care of you. |
| 1. **Assess Physical Violence** | Make patients more likely to disclose by using specific examples of violence and unwanted sex. Patients are less likely to disclose when you use phrases like “rape” “abuse” or “domestic violence” | Have you ever been in a relationship where your partner has hit, pushed, or slapped you?  Have you ever been in a relationship where your partner threatened you with violence?  Have you ever been in a relationship where your partner has thrown, broken, or punched things?^1^ |
| 1. **Assess Sexual Violence** |  | Has anyone ever made you have intercourse, oral or anal sex against your will?  Has anyone ever touched private parts of your body, or made you touch theirs, under force or threat?  Has anyone ever taken advantage of you sexually when you were too drunk or out of it to stop it?  Have there any other situations in which another person tried to force you to have unwanted sexual contact?^2^ |
| **If the patient endorses ANY item from Step 2 or Step 3, proceed to steps 4 and 5.** | | |
| 1. **Empathize** | Validate the seriousness of abuse and the difficulty of disclosure by providing the patient with empathy. | That must have been difficult to talk about. Thank you for trusting me with this information. |
|  |  | IT IS NOT YOUR FAULT that someone hurt you. No one deserves to be treated that way. |
|  |  | You deserve to be treated with respect in all relationships. You especially deserve to feel safe and comfortable. I am concerned and would like to help. |
| 1. **Follow-up** | Provide patient with specific options for addressing trauma through medical care, mental health care, and social resources | Our clinic offers programs to help women with these experiences  [INSERT CONTACT INFO] |
|  |  | Our medical team includes psychologists who work with women who have experienced violence. They can help you cope with emotions and memories related to this experience.  [INSERT CONTACT INFO] |
|  |  | Your health care should be the least scary and most comfortable experience possible. A lot of women find aspects of their OB/GYN care uncomfortable, painful, or frightening. What parts of the visit could I make more comfortable for you? |

References

1. Paranjape, A., Rask, K., & Liebschutz, J. (2006). Utility of STaT for the identification of recent intimate partner violence. Journal of the National Medical Association, 98(10), 1663-1669.
2. Koss, M. P., Abbey, A., Campbell, R., Cook, S., Norris, J., Testa, M., . . . White, J. (2008). Revising the SES: A collaborative process to improve assessment of sexual aggression and victimization (psychology of women quarterly (2007) 31, (357-370)). Psychology of Women Quarterly, 32(4), 493. doi:10.1111/j.1471-6402.2008.00468.x
