# Supplementary material for: Assessing Trauma History in Pregnant Patients: A Didactic Module and Role-Play for Obstetrics and Gynecology Residents
Source: MedEdPORTAL. 2020 Jul 20;16:10925. doi: 10.15766/mep_2374-8265.10925 (PMC7373354; doi:10.15766/mep_2374-8265.10925)
Supplement: Supplementary file 1 — Didactic Facilitator Guide.docxPowerPoint Slides.pptxHandout 1 Sample Chart of Pregnant Patient With PTSD.docxHandout 2 Communication Template.docxHandout 3 Sample Trauma-Informed Practice.docxHandout 4 Sample Trauma Narrative for Role-Play.docxPocket Guide for Trauma History Screening.pdfAssessment Tool.docx [file mep_2374-8265.10925-s001.zip › E. Handout 3 Sample Trauma-Informed Practice.docx]

Handout #3: “Sample Trauma-informed Questions, Phrases, and Statements for Practice” (Appendix E)

**WHAT TO SAY**:

1. “It is important that we conduct a portion of our evaluation with just you and me in order to ensure your privacy. Your [friend, partner, family member] is most welcome to return after stepping out for this part. Do you have any questions or concerns about this part of our routine?”

2. “Violence is a major problem that has serious effects on our health. I am going to ask you some questions about some of these experiences.”

3. “These questions are very personal, so I need to make it clear that this information is confidential, meaning that it will only be shared with people who are directly involved with your medical care. Do you have any questions about this?”

4. “Unfortunately, most women have had experiences with violence or emotional mistreatment.”

5. “Have you ever been hit, slapped, or otherwise physically hurt by someone? Has this happened in your relationship with your current partner?”

6. “Has anyone ever forced you to engage in sexual activities when you didn’t want to? Has this happened in your relationship with your current partner?”

7. Have you ever felt pressured to engage in certain sexual activities that you were not comfortable with?

8. Have you ever been forced to have unwanted sex while you were unable to stop it? (i.e., if you were unconscious or too “out of it”)

9. “Thank you for trusting me with this information.”

10. “It is not your fault when someone forces you to have sex when you don’t want to. No one deserves to be treated that way.”

11. “It doesn’t matter whether the person who hurt you was a family member, friend, or significant other. It doesn’t matter where you were, if you drank too much, or were unconscious. The only person responsible for assaulting you is *the person who assaulted you.”*

12. “How are you coping with these experiences? Are you ever bothered by bad memories? Do you ever feel anxious or afraid when you think about them?”

13. “Are there situations that make you feel anxious or uncomfortable, such as medical exams?”

14. “It also sounds as if you have some uncomfortable feelings, like anxiety. This is very common when people have experienced violence, and it is a very serious problem that *can* affect the health of your pregnancy.”

15. “You deserve to be treated with respect in all relationships, and you especially deserve to feel safe and comfortable. I am concerned for you and your baby’s health and would like to help.”

16. “Part of our women’s health team includes trained psychologists who specialize in helping pregnant women who have experienced violence, or who feel worried, tense, irritable, or anxious. *You* are an important part of this team.

17. “Your health care should be the *least* scary and *most* comfortable experience possible. A lot of women find aspects of prenatal care uncomfortable, painful, or even frightening. What are the least comfortable parts of a physical exam for you?”

18. “I read in your chart that you have been seeing a counselor for therapy. What are you and the therapist working on together?”

19. “I noticed in your chart that you have a counselor but haven’t seen him/her in a while. What prompted you to stop attending sessions?”

20. “What strategies has your counselor given you to help with emotional difficulties, like anxiety or depression?”

**WHAT NOT TO SAY**:

1. “You haven’t had any issues with trauma, right?” (Leading question)

2. “Any trauma or abuse?” (Not a full question; semi-leading; vague terminology)

3. “Have you ever been raped?” (Vague terminology; open to various interpretations)

4. “Has anyone ever molested you?” (Vague terminology; open to various interpretations)

5. “Has anyone ever touched you inappropriately?” (Vague terminology; open to various interpretations)

6. “Have you experienced domestic violence?” (Vague terminology; open to various interpretations)

7. “Any issues with abuse you want to talk about?” (See #2; patient may not want to talk about it and still appreciate the opportunity to disclose)

8. “I know how you feel.” (Generally, self-disclosure is best avoided as providers don’t necessarily “know” how a patient is feeling.)

9. “Why didn’t you report it/tell someone?” (Victim-blaming)

10. “Were you drunk/intoxicated? What were you doing/wearing at the time?” (Victim-blaming; It is not provider’s responsibility to determine veracity of the patient’s disclosure).

**REMEMBER: SCREENING FOR TRAUMA REQUIRES A COMBINATION OF OPEN- AND CLOSED-ENDED QUESTIONS TO GATHER ACCURATE INFORMATION FROM THE PATIENT. AVOID LEADING QUESTIONS AND NONSPECIFIC PHRASES. THESE ARE LEAST LIKELY TO ELICIT ACCURATE RESPONSES**

**PRACTICE PHRASES TO USE DURING OBSTETRIC EXAMS/PROCEDURES**

1. “I won’t go ahead with any part of the procedure until you tell me you are ready.”
2. “Sometimes it can be hard to tell someone that you are uncomfortable. What is the best way for you to tell me when you need me to stop the exam? People sometimes say, ‘Wait’ or ‘I need a second’ or ‘Stop please.’ Why don’t you try saying it now.”
3. “The most important thing is for me to know that you are doing okay.”
4. “I notice that you are tense/upset. I am stopping what I am doing.”
5. “These procedures can be more uncomfortable when you are tense or upset. What is the best way for you to be comfortable?”
6. “If there is risk to you or your baby, I may need to perform a procedure that causes discomfort. We will do everything we can to make you as comfortable as possible. It is important that you let us know how you are doing.”
