# Supplementary material for: Assessing Trauma History in Pregnant Patients: A Didactic Module and Role-Play for Obstetrics and Gynecology Residents
Source: MedEdPORTAL. 2020 Jul 20;16:10925. doi: 10.15766/mep_2374-8265.10925 (PMC7373354; doi:10.15766/mep_2374-8265.10925)
Supplement: Supplementary file 1 — Didactic Facilitator Guide.docxPowerPoint Slides.pptxHandout 1 Sample Chart of Pregnant Patient With PTSD.docxHandout 2 Communication Template.docxHandout 3 Sample Trauma-Informed Practice.docxHandout 4 Sample Trauma Narrative for Role-Play.docxPocket Guide for Trauma History Screening.pdfAssessment Tool.docx [file mep_2374-8265.10925-s001.zip › F. Handout 4 Sample Trauma Narrative for Role-Play.docx]

Handout #4: “Sample Trauma Narrative for Role-Play”

| **Simulated OB Patient Narrative**  **Instructions for Role-Play (Simulated Patient/Facilitator)**: Your delivery of responses should be flat and quiet. Do not offer any more information than what is asked. Avoid making much eye contact with the provider. | |
| --- | --- |
| **Topic/Question** | ***Narrative Response*** |
| **Hx sexual trauma?** | *Yeah, in high school. It was my brother’s friend, my boyfriend’s friend, too. But I don’t talk to any of them anymore, so it’s fine.* |
| **If asked follow-ups:** | - *I was 14.* - *It was at a party.* - *They were my older brother’s friends.* - *There were four of them.* - *I only ever told my mom.* - *Mom asked me why I wasn’t more hurt if it was really rape.* |
| **Other traumas?** | *Nothing like that* [the gang rape]*.* |
| **Other unwanted sex?** | *What do you mean?* |
| **Clinician elaborates about someone. . .**   - **Pressuring you** - **Wearing you down** - **Making threats (like ending the relationship)** - **Making false promises** - **Giving you alcohol/drugs** - **Taking advantage of your level of intoxication** - **Etc.** | - *One of the guys* {from the gang rape] *wasn’t as bad. We started going out later that year. He was the only one who said nice things to me at the time and tried to make it not so bad. But later he always tried to get me to do things I didn’t really want to.* - *He would get me to do things with him when my brother and his friends were around in the house. A couple times they were in the same room.* - *Once I know someone gave me something at a party because I don’t remember anything after that and I didn’t have that much to drink. I know that my brother’s friends did things with me because I saw pictures of it later.* - *When I was in college, there were a lot of times when guys would make me have sex when I didn’t want to. They never actually hurt me, though, and sometimes we were technically dating so I guess it wasn’t really that bad.* |
| **Physical Violence?** | - *My boyfriend in high school used to hit me.* - *It wasn’t usually that big a deal – he would just slap me sometimes or grab me hard.* - *It got pretty bad when he found out I was pregnant the first time, though. He beat me up and forced me to get an abortion.* - *My boyfriend now doesn’t hurt me.* |
| **Current sexual functioning? Follow-ups?** | - *My boyfriend and I have sex pretty much whenever he wants it. Most of the time it’s when my son is home because of our work schedules. I don’t like that cuz the walls are pretty thin, but. . . I mean, I just go somewhere else in my head.* - *It hurts a lot of the time. I usually don’t feel like doing it, but my boyfriend is a nice guy. He deserves it. I mostly try to just go somewhere else in my head.* - *I wish he would use condoms, cuz I know he had an STD before. But he doesn’t like to, so I mostly give in.* |
| **Depression?** | *I’m doing fine. I don’t need any therapy or medication, if that’s what you mean.* |
| **Have you told anyone else about these experiences? Family support?** | *Not really. Like I said my mom didn’t really believe me, she thought I was complaining because I didn’t look hurt. I live with my grandma. I try not to go around those family member who you know still do things like they drink a lot, party a lot. I try not to mess with that anymore.* |
