# Supplementary material for: Assessing Trauma History in Pregnant Patients: A Didactic Module and Role-Play for Obstetrics and Gynecology Residents
Source: MedEdPORTAL. 2020 Jul 20;16:10925. doi: 10.15766/mep_2374-8265.10925 (PMC7373354; doi:10.15766/mep_2374-8265.10925)
Supplement: Supplementary file 1 — Didactic Facilitator Guide.docxPowerPoint Slides.pptxHandout 1 Sample Chart of Pregnant Patient With PTSD.docxHandout 2 Communication Template.docxHandout 3 Sample Trauma-Informed Practice.docxHandout 4 Sample Trauma Narrative for Role-Play.docxPocket Guide for Trauma History Screening.pdfAssessment Tool.docx [file mep_2374-8265.10925-s001.zip › G. Pocket Guide for Trauma History Screening.pdf]

5. Follow-up

There are programs to help women with these experiences deal with stress during their pregnancies.
[Insert contact information]

There are trained counselors/therapists who work with women who have experienced violence. They can help you cope with emotions and memories related to this experience.
[Insert referral information]

A lot of women find aspects of their OB/GYN care uncomfortable, painful, or frightening. Your health care should be the least scary and most comfortable experience possible. What parts of the visit could I make more comfortable for you?

For other resources or if you have any questions, please contact [*insert contact information*]

Screening for Interpersonal Violence

A TO-CARE Guide

Trauma-informed Obstetric Care

1. Build Trust

Many women have had experience with violence. I am going to ask you some questions about violence, since these experiences can affect your health.

It is important that we conduct part of your visit with just you in order to ensure your privacy. Would you mind if your[friend, partner, family member] stepped out for a moment?

Answering questions about violence may be uncomfortable, but knowing what you've been through helps me take better care of you.

2. Assess Physical Violence

Have you ever been in a relationship where your partner has hit, pushed, or slapped you?

Have you ever been in a relationship where your partner threatened you with violence?

Have you ever been in a relationship where your partner has thrown, broken, or punched things?

3. Assess Sexual Violence

Has anyone ever made you have intercourse, oral or anal sex against your will?

Has anyone ever touched private parts of your body, or made you touch theirs, under force or threat?

Has anyone ever taken advantage of you sexually when you were too drunk or out of it to stop it?

Have there been any other situations in which another person tried to force you to have unwanted sexual contact?

! If the patient endorses ANY item from Step 2 or 3, proceed to Steps 4 and 5.

4. Empathize

That must have been difficult to talk about. Thank you for trusting me with this information.

IT IS NOT YOUR FAULT that someone hurt you. No one deserves to be treated that way.

You deserve to be treated with respect in all relationships. You especially deserve to feel safe and comfortable. I am concerned and would like to help.
