# Supplementary material for: Assessing Trauma History in Pregnant Patients: A Didactic Module and Role-Play for Obstetrics and Gynecology Residents
Source: MedEdPORTAL. 2020 Jul 20;16:10925. doi: 10.15766/mep_2374-8265.10925 (PMC7373354; doi:10.15766/mep_2374-8265.10925)
Supplement: Supplementary file 1 — Didactic Facilitator Guide.docxPowerPoint Slides.pptxHandout 1 Sample Chart of Pregnant Patient With PTSD.docxHandout 2 Communication Template.docxHandout 3 Sample Trauma-Informed Practice.docxHandout 4 Sample Trauma Narrative for Role-Play.docxPocket Guide for Trauma History Screening.pdfAssessment Tool.docx [file mep_2374-8265.10925-s001.zip › H. Assessment Tool.docx]

**Assessment of Beliefs, Awareness, and Sense of Efficacy in Trauma-Informed Care**

**Questionnaire adapted from:**

1. Kassam-Adams N, Rzucidlo S, Campbell M, Good G, Bonifacio E, Slouf K, Schneider S, McKenna C, Hanson CA, Grather D. Nurses' views and current practice of trauma-informed pediatric nursing care. J Pediatr Nurs. 2015;30(3):478-84.
2. Bruce MM, Kassam-Adams N, Rogers M, Anderson KM, Sluys KP, Richmond TS. Trauma providers' knowledge, views, and practice of trauma-informed care. J Trauma Nurs 2018;25(2):131-8.

**PART 1: Beliefs about Trauma-Informed care**

Please use the scale below to indicate how much you agree/disagree with the following statements:

|  |  | **Strongly Disagree** | **Disagree** | **Agree** | **Strongly Agree** |
| --- | --- | --- | --- | --- | --- |
| 1. | Providers should focus on physical health for patients as opposed to patients’ mental health. | 1 | 2 | 3 | 4 |
| 2. | The way that medical exams are provided can be changed to make it less stressful for patients. | 1 | 2 | 3 | 4 |
| 3. | Medical providers can teach patients how to cope with trauma. | 1 | 2 | 3 | 4 |
| 4. | Medical providers should regularly assess for symptoms of traumatic stress. | 1 | 2 | 3 | 4 |
| 5. | It is necessary for medical providers to have mental health information about their patients in order to provide appropriate medical care. | 1 | 2 | 3 | 4 |

**PART 2: Awareness:** Circle how much you agree with the following statements.

|  |  | **Strongly Disagree** | **Disagree** | **Agree** | **Strongly Agree** |
| --- | --- | --- | --- | --- | --- |
| 1. | A significant portion of patients have experienced trauma and/or abuse in their lifetime | 1 | 2 | 3 | 4 |
| 2. | It is inevitable that most patients who experience trauma and/or abuse will go on to develop significant posttraumatic stress or PTSD. | 1 | 2 | 3 | 4 |
| 3. | Many patients cope well on their own after experiencing trauma and/or abuse. | 1 | 2 | 3 | 4 |
| 4. | Patients with significant posttraumatic stress reactions usually show obvious signs of distress. | 1 | 2 | 3 | 4 |
| 5. | There is a common profile for the PTSD patient. | 1 | 2 | 3 | 4 |
| 6. | There are things I can do for patients with trauma and/or abuse history to help prevent worsening or recurring symptoms during medical exams | 1 | 2 | 3 | 4 |
| 7. | There are effective screening measures for assessing traumatic stress that we can use in practice. | 1 | 2 | 3 | 4 |
| 8. | The psychological effects of trauma and abuse often persist after physical injuries are healed. | 1 | 2 | 3 | 4 |
| 9. | I understand that traumatic stress may present itself differently (i.e., some patients may be angry and irritable, others are “shut-down,” and others are fearful) | 1 | 2 | 3 | 4 |
| 10. | There is lacking empirical evidence supporting assessment and intervention for traumatic stress | 1 | 2 | 3 | 4 |
| 11. | It would be harmful for a patient to be forced to acknowledge their trauma | 1 | 2 | 3 | 4 |

**PART 3: Sense of Efficacy:** Rate how competent you feel in using each skill below.

|  |  | **Not competent** | **Somewhat competent** | **Very competent** |
| --- | --- | --- | --- | --- |
| 1. | Engaging with traumatized patients so that they feel comfortable talking to you/ comforted by you | 1 | 2 | 3 |
| 2. | Responding calmly and without judgment to a patient’s strong emotional distress | 1 | 2 | 3 |
| 3. | Eliciting details of a trauma and/or abuse from a patient without re-traumatizing them | 1 | 2 | 3 |
| 4. | Educating patients about common traumatic stress reactions and symptoms | 1 | 2 | 3 |
| 5. | Avoiding or altering situations within the hospital that a patient might experience as traumatic | 1 | 2 | 3 |
| 6. | Responding to a patient’s question about whether their traumatic stress reactions will ever get better | 1 | 2 | 3 |
| 7. | Assessing a patient’s distress, emotional needs, and support systems soon after traumatic or abuse | 1 | 2 | 3 |
| 8. | Recognizing NONverbal signs of emotional distress or pain, e.g. muscle tensing, facial expression, patient crying, etc. | 1 | 2 | 3 |
| 9. | Eliciting a coping strategy that will help the patient be more comfortable during invasive exams or procedures | 1 | 2 | 3 |
| 10. | Providing verbal guidance to patients during procedures (e.g. now take a deep breath…) | 1 | 2 | 3 |
| 11. | Providing feedback to patients during procedures (e.g. great job.. release the tension in your muscles a little more next time…) | 1 | 2 | 3 |
| 12. | Explaining each step of the exam or procedure before you move on. | 1 | 2 | 3 |
| 13. | Instructing others in the room to help patient cope? | 1 | 2 | 3 |
